# More than Just Two Sexes: The Neural Correlates of Voice Gender Perception in Gender Dysphoria

**DOI:** 10.1371/journal.pone.0111672

**Published:** 2014-11-06

**Authors:** Jessica Junger, Ute Habel, Sabine Bröhr, Josef Neulen, Christiane Neuschaefer-Rube, Peter Birkholz, Christian Kohler, Frank Schneider, Birgit Derntl, Katharina Pauly

**Affiliations:** 1 Department of Psychiatry, Psychotherapy and Psychosomatics, Medical School, RWTH Aachen University, Aachen, Germany; 2 Jülich Aachen Research Alliance-Translational Brain Medicine, Jülich, Germany; 3 Department of Gynaecological Endocrinology and Reproductive Medicine, Medical School, RWTH Aachen University, Aachen, Germany; 4 Department of Phoniatrics, Pedaudiology and Communication Disorders, Medical School, RWTH Aachen University, Aachen, Germany; 5 Department of Psychiatry, Neuropsychiatry Division, University of Pennsylvania School of Medicine, Philadelphia, Pennsylvania, United States of America; University of Goettingen, Germany

## Abstract

Gender dysphoria (also known as “transsexualism”) is characterized as a discrepancy between anatomical sex and gender identity. Research points towards neurobiological influences. Due to the sexually dimorphic characteristics of the human voice, voice gender perception provides a biologically relevant function, e.g. in the context of mating selection. There is evidence for a better recognition of voices of the opposite sex and a differentiation of the sexes in its underlying functional cerebral correlates, namely the prefrontal and middle temporal areas. This fMRI study investigated the neural correlates of voice gender perception in 32 male-to-female gender dysphoric individuals (MtFs) compared to 20 non-gender dysphoric men and 19 non-gender dysphoric women. Participants indicated the sex of 240 voice stimuli modified in semitone steps in the direction to the other gender. Compared to men and women, MtFs showed differences in a neural network including the medial prefrontal gyrus, the insula, and the precuneus when responding to male vs. female voices. With increased voice morphing men recruited more prefrontal areas compared to women and MtFs, while MtFs revealed a pattern more similar to women. On a behavioral and neuronal level, our results support the feeling of MtFs reporting they cannot identify with their assigned sex.

## Introduction

The dichotomous classification of gender is based on the fact that the two sexes are essential to the survival of our [1] and other [2] species. A combination of chromosome complement and genital and gonadal phenotypes also contributes to the conventional dichotomy [3]. However, non-binary gender diversity abounds in both humans and animals [4]. Primary sexual characteristics such as testes and ovaries develop in early pregnancy prior to the sexual differentiation of the brain. The latter is associated with sexually distinctive behavior and appears to be continuously influenced by sex hormones. The two processes are independent of each other and can take different routes [5]. Therefore, increasing evidence is linking their disparate differentiation to gender dysphoria [6] also known as transsexualism [7], [8]. According to DSM-5, gender dysphoria describes the experience of a marked incongruence between one's assigned and one's expressed gender [9]. Gender denotes one's public role as male or female with biological and psychological factors interacting with gender development. Expressed gender includes several alternative identities beyond binary stereotypes [10]. This discrepancy, accompanied by distress or impairment in social and occupational functioning, can result in mental problems calling for clinical interventions [11], [12], [13]. Individuals who suffer from gender dysphoria often aspire to adapt their assigned to their expressed gender with hormonal treatment or sex-reassignment surgery [14]. Those individuals are often referred to with assigned-to-target sex terms such as male-to-female (MtFs) or female-to-male (FtMs).

Although the etiopathogenesis of gender dysphoria is yet to be adequately defined, recent findings point towards neurobiological mechanisms involved [15]. No detectable influences of hormonal status [16] on gender dysphoria have been found so far. Concerning genetic factors the evidence is inconsistent: Some studies lacked to find genetic influences [17], [18], [19], whereas Hare and colleagues [20] identified an association between gender dysphoria and the androgen receptor allele. This is consistent with studies suggesting a much higher prevalence of gender dysphoria in monozygotic than in dizygotic twins [21] as well as in non-twin siblings than in the general population [22]. The experience of gender identity is linked to the sexual differentiation of the brain and may therefore be detectable in brain structure and function [8], [23]. Post-mortem anatomical studies have consistently revealed a similarity in the bed nucleus of stria terminalis (BSTc), one of the regions with marked gender differences in volume, between MtFs and women [24], [25] as well as between one FtM and men [26]. However, generalization from these findings is limited by the constraints of small sample sizes in post-mortem studies.

In contrast, magnetic resonance imaging (MRI) is a sensitive tool to investigate the cerebral basis of sexual differentiation in vivo. There are only a few structural studies examining gender dysphoria: Simon and colleagues [27] reported grey matter volume differences between gender dysphoric individuals and a mixed gender control group, Luders and colleagues [28] as well as Savic and Arver [29] provided evidence for differences in grey matter volumes between individuals with gender dysphoria and both men and women. Rametti and colleagues [30] reported an intermediate position for hormonally untreated MtFs between male and female brains by measure of white matter microstructure. Further, studies found female-like white matter structures in FtMs [31] as well as changes in white matter structure in FtMs [32], [33] and cortical thickness in FtMs and MtFs [33] through hormonal treatment. Thus, structural evidence points towards differences between gender dysphoric individuals and their biological and partly also the opposite sex as well as changes through the intake of hormones.

Functional imaging provides a more directed pattern: Greater similarity was found between an untreated FtM and females compared to males in functional connectivity maps involving the lingual gyrus and the precuneus [34]. However, in the light of a single case study, results should be taken with caution. In contrast, to the best of our knowledge other functional studies, so far, rather found male-like brain activation in FtMs [35] and female-like cerebral activity in MtFs in language and mental rotation tasks [36], [37], [38] as well as during the presentation of erotic stimuli [39] and, therefore, in tasks especially sensitive to neural sex differences in neuronal activity [40], [41], [42]. Thus in sum, findings from functional studies point toward increased similarities between gender dysphoric individuals and their aspired gender. However, the amount of research that has been done in this area is rather small, and differences in sample size and methodology have led to an ambiguous integration of gender dysphoria into neurobiological research. Further studies with larger sample sizes are needed to explore additional biological and social factors influencing gender-specific experience and behavior [43], [44].

The human voice is an important secondary sexual characteristic directly linked to gender identification. It plays a key role in social interaction [45], [46] and mate selection [47], [48], [49]. The extra-linguistic parameters, fundamental frequency (F0; i.e. vocal fold vibration perceived as pitch) and formant frequencies (i.e. vocal tract resonances) are of major relevance to sex identification [50], [51], [52], [53]. While the average voice pitch varies about one octave (i.e. 12 semitones) between men and women, there is an overlapping, “gender-ambiguous” range in which the decision for a male or female voice depends especially on formants and other contextual parameters such as visual information or prosodic characteristics [51], [54], [55].

Voices are first processed in the superior temporal gyri, the anterior superior temporal sulci and the middle temporal gyri [46], [56], [57], [58], [59], [60]. Further there is evidence for an involvement of the inferior frontal gyrus and the cerebellum in voice gender perception [61]. Thereby right inferior frontal gyrus seems to reflect the processing of F0 modulation, which is the main acoustic correlate of prosody [62]. In a male sample, the differentiation of male and female voices resulted in greater activation in the right anterior superior temporal gyrus while listening to female voices, and in the right precuneus while perceiving male voices [52]. In a gender-mixed sample, stronger neural activity was observed in response to female as compared to male voices in the right supratemporal plane, the right posterior superior temporal gyrus, left postcentral gyrus, the bilateral inferior parietal lobes and the insula [63]. In a previous study [64], we found the first evidence of an interaction between voice gender and the listener's sex; namely, stronger activation in men compared to women in the left middle temporal, left orbital and right medial prefrontal cortices for the processing of female compared to male voices. Further, increased voice gender morphing in the direction of the opposite sex resulted in stronger activation in the superior and middle frontal gyri in men compared to women.

A possible explanation for this may come from animal research, which indicated a neural basis for sex differences in the auditory perception of mating calls [65], [66]. Detection of gender distinguishing signals in general is important for the identification of adequate mates and results in a preference as well as stronger emotional and attentional reactions to signals of the opposite sex [67], [68], [69]. Thus, sexual dimorphism of vocalization and its perception in both animals and humans are evolutionary relevant for reproductive needs. Therefore, we expected that a voice gender perception task should be highly sensitive to sex differences in neuronal activity in areas involved in auditory, affective, attentional and evaluative processes.

In the present study, we aimed at investigating the neural correlates of voice gender processing in MtFs in comparison to men and women. Based on the few structural and functional findings, so far, we speculate to find at least distinct neural activation in MtFs compared to men and women if not a tendency to a more female-like activation pattern in voice-selective brain regions, such as the superior and middle temporal gyri and prefrontal areas. Further, we hypothesized that hormonally treated (contrasted to untreated) MtFs are more equal to the aspired sex. Results are discussed with respect to an integration of gender-variant identities into our understanding of the sexes.

## Materials and Methods

### Participants

17 hormonally untreated MtFs, 16 hormonally treated MtFs, 21 non-gender dysphoric men and 20 non-gender dysphoric women participated in this study. MtFs were recruited in self-help groups at the Department of Phoniatrics, Pedaudiology and Communication Disorders of the RWTH Aachen University Hospital and by word-of-mouth recommendation. MtFs identified themselves as gender dysphoric, expressed a strong sense of belonging to the opposite sex, and lived the desired role in everyday life. MtFs taking hormones were treated following the German transsexual law [70] for at least 3 months and therefore had overcome the first phase of endocrinological adjustment. Untreated MtFs declared their intention of undergoing cross-sex hormone therapy in the future. The German version of the Structured Clinical Interview of the fourth edition of the Diagnostic and Statistical Manual of Mental Disorders (DSM-IV) [71] was used to ensure the exclusion of participants with mental disorders of axis I unrelated to gender dysphoria. Further exclusion criteria were neurological disorders and other medical conditions affecting the cerebral metabolism as well as first degree relatives with a history of mental diseases. All participants were native speakers of German and right-handed aside from one left-handed participant in each group. Handedness was assessed by means of the Edinburgh Handedness Inventory [72].

The hormonal status was obtained on the day of testing from all participants, except 3 from whom no blood samples could be taken and 5 from whom some blood parameters were not available due to technical problems. The number of hetero- and homosexual participants was equal in both MtF samples. (Sexual orientation in MtFs was defined according to their anatomical sex, i.e. homosexual MtFs prefer male partners).

Four participants were excluded due to excessive movement in the scanner. Hence, data from 71 participants (16 untreated MtFs, 16 treated MtFs, 19 women, 20 men) were included in the final analyses (Table 1). Data pertaining to the controls have already been reported [64]. Kolmogorov-Smirnov tests showed that data were normally distributed regarding age, years of education or crystallized verbal intelligence [73] estimation. Using analyses of variance (ANOVA) group comparisons showed no significant differences in all three measures. Since hormonal data did not fulfill the assumptions of normal distribution, nonparametric Kruskal-Wallis tests were used for analyzing the latter (Table 1).

**Table 1:** Characteristics of the sample (mean and standard deviations for age, education, IQ, hormonal level and sexual orientation) and group comparisons.

	Men	Women	MtF untreated	MtF treated	*P (ANOVA)*
**Age**	32.35 (10.27)	33.16 (12.34)	36.38 (14.02)	30.19 (10.95)	0.528
**Education**	15.00 (2.92)	14.95 (3.21)	14.50 (3.06)	13.81 (3.04)	0.646
**IQ**	112.45 (14.72)	112.21 (16.14)	113.13 (13.57)	104.25 (6.81)	0.204
**Sexual orientation** D					***P (Chi square)***
Heterosexual	18	18	7	5	
Homosexual	1	0	7	9	
Bisexual	0	0	2	2	
					<0.001*
**Hormonal level**					***P (Kruskal-Wallis)***
17-ß-Estradiol (pmol/l)	88.62 (34.89)	136.04 (134.52)	91.67 (50.92)	1400.10 (3137.44)^A^	<0.001*
FSH (U/l)	5.35 (6.08)	13.4 (24.97)	4.62 (2.77)	5.57 (9,99)	0.055
LH (U/l)	5.76 (1.99)	10.32 (15.26)	5.30 (3.08)	4.14 (9.72)	0.011
Progesterone (nmol/l)	2.32 (1.01)	4.46 (8.69)	2.06 (1.04)	1.7 (0.80)	0.267
Prolactin (mU/l)	166.16 (58.41)	195.42 (57.12)	165.50 (65.25)	571.46 (538.28)^A^	0.001*
Sex steroid binding globulin (SHBG; nmol/l)	27.88 (11.05)^C^	127.83 (65.95)^B^	40.28 (40.01)^C^	91.19 (71.76)	<0.001*
Free testosterone (pmol/l)	40.71 (14.32)^C^	3.74 (2.33)^B^	32.85 (13.64)^C^	4.48 (5.97)	<0.001*

Significant differences are marked in asterisk.

A significant difference with respect to all three other groups, *p* = 0.008 Bonferroni corrected.

B significant differences with respect to men and MtF untreated, *p* = 0.008 Bonferroni corrected.

C significant differences with respect to MtF treated, *p* = 0.008 Bonferroni corrected.

D respective data are missing in one man and one woman.

The local Institutional Ethics Committee of the Medical Faculty of RWTH Aachen University approved the study (reference: EK 088/09). All participants were financially reimbursed and gave their written informed consent.

### Stimuli and procedure

A more detailed description of the stimulus preparation and presentation appears in Junger et al. [64]. In brief, the voices of 10 men and 10 women were recorded while reading 3 out of 6 emotionally neutral 3-syllable nouns. To guarantee a natural and consistent prosody and pronunciation, target words were spoken in the context of the carrier sentence “I said …” and then cut out subsequently. The resulting 30 original male and 30 original female voice stimuli were further modified (morphed) in 2, 4 and 6 semitone steps (st) in the direction of the other gender. The software Praat [74] was used to shift pitch contour and formant structure accordingly. Correspondingly, the final task consisted of 8 experimental conditions in a 2×4 event-related design with the factors voice gender and morphing level: 1.) original/0st male voice, 2.) male voice morphed by 2st, 3.) male voice morphed by 4st, 4.) male voice morphed by 6st, 5.) original/0st female voice, 6.) female voice morphed by 2st, 7.) female voice morphed by 4st, and finally 8.) female voice morphed by 6st. Every condition was presented ten times and comprised 3 different nouns spoken by 3 different voices of the same sex. This resulted in a total of 240 voice stimuli presented in a pseudo-randomized order.

Stimulus presentation was done via electrostatic headphones with Presentation 14.2 software (http://www.neurobs.com) individually adapted for loudness. Participants were asked to indicate the gender of each speaker by button press as fast as possible.

### Analysis of the behavioral data

Behavioral data were analyzed using SPSS 18.0.0 (SPSS Inc., Chicago, IL). Since data were not normally distributed, nonparametric Friedman rank tests [75] were performed to detect mean rank differences between conditions regarding the amount of hits and reaction times (RT). Due to the two-alternative discrimination task, inclusion of the error rates only revealed a pattern opposite to the hit rates and thus provided no additional information.

To decompose significant effects regarding voice gender (male/female) and stimulus morphing (0st/2st/4st/6st) for hits and RT, Wilcoxon tests were calculated for within-group comparisons in the whole group and each group separately and Mann-Whitney-U tests for the between-groups comparisons.

For each morphing level, discrimination sensitivity (d-prime - d') and potential response biases (log ß) [76] were evaluated for two-alternative discrimination tasks [77]. Because of their normal distribution, one-sample t-tests were calculated for each group separately in order to analyze if d-prime was above chance level and if potential biases reached significance. To compare groups, ANOVAs were computed and decomposed by post hoc two-sample t-tests where significant.

All post-hoc tests were Bonferroni corrected for multiple comparisons.

All data points used to determine averages and summary statistics in Table 1 and Table 2 as well as in the behavioral results section can be found in Table S4–Table S7 in File S1.

**Table 2:** Behavioral outcome measures.

	Men	Women	MtF	Men	Women	MtF
	Mean (SD)	Mean (SD)	Mean (SD)	Mean (SD)	Mean (SD)	Mean (SD)
	***Hits (%)***					
	***Male voice***			***Female voice***		
**0st**	97.00 (3.73)	99.29 (1.78)	98.02 (2.79)	97.66 (2.88)	94.21 (6.29)	93.44 (7.83)
**2st**	90.66 (7.48)	96.84 (4.07)	93.33 (7.43)	91.16 (10.83)	80.35 (11.75)	87.19 (9.20)
**4st**	63.00 (15.02)	83.15 (12.78)	75.94 (12.64)	81.66 (9.64)	65.78 (15.98)	72.19 (11.87)
**6st**	36.50 (17.18)	62.63 (15.65)	52.19 (17.47)	58.66 (16.27)	36.49 (15.93)	50.94 (15.48)
	***Reaction time***					
	***Male voice***			***Female voice***		
**0st**	1.22 (0.19)	1.06 (0.15)	1.14 (0.18)	1.25 (0.23)	1.07 (0.16)	1.18 (0.2)
**2st**	1.32 (0.29)	1.14 (0.14)	1.25 (0.22)	1.32 (0.27)	1.18 (0.24)	1.32 (0.22)
**4st**	1.51 (0.29)	1.22 (0.13)	1.39 (0.23)	1.34 (0.25)	1.27 (0.21)	1.32 (0.21)
**6st**	1.65 (0.37)	1.35 (0.16)	1.53 (0.30)	1.47 (0.29)	1.38 (0.27)	1.39 (0.24)
	***D-prime***			***Log β***		
**0st**	3.87 (0.34)	3.78 (0.41)	3.58 (0.55)	−0.14 (0.74)	0.63 (0.75)	0.49 (0.84)
**2st**	3.03 (0.48)	2.84 (0.40)	2.88 (0.58)	−0.20 (1.29)	1.22 (1.08)	0.57 (1.07)
**4st**	1.35 (0.32)	1.53 (0.43)	1.40 (0.39)	−0.36 (0.53)	0.37 (0.49)	0.05 (0.49)
**6st**	−0.15 (0.45)	−0.03 (0.29)	0.09 (0.42)	−0.07 (0.30)	0.37 (0.49)	0.03 (0.21)

Mean percentage of hits and reaction times (in seconds) for correct responses, discrimination sensitivity (d-prime) and answering bias (log ß) in response to male and female voices of the different morphing steps in semitones (st) for men, women and MtFs.

### fMRI data acquisition and pre-processing

Functional imaging data were acquired at the Department of Psychiatry, Psychotherapy and Psychosomatics of the RWTH Aachen University Hospital on a 3 T Siemens Trio MR Scanner (Siemens Medical Systems, Erlangen, Germany). Echo-planar imaging (EPI) sensitive to blood-oxygen-level-dependent (BOLD) contrast were used (T2*, voxel size: 3.1×3.1×3.1 mm^3^, distance factor 15%, GAP 0.5 mm, 64×64 matrix, FoV: 200×200 mm^2^, TR = 2s, TE = 30 ms, α = 76°) with 36 slices covering the entire brain. To avoid magnetic field saturation effects, image acquisition was preceded by 6 dummy scans which were discarded before preprocessing. The resulting 785 volumes per subject were analyzed using SPM8 (http://www.fil.ion.ucl.ac.uk/spm) implemented in MATLAB 2010b (Mathworks, Sherborn, MA). Images were realigned to the first volume. Spatial normalization into MNI space was accomplished by means of the unified segmentation approach [78]; an 8 mm FWHM Gaussian kernel was used for smoothing. A 128 Hz high pass filter removed effects of low frequency noise.

### Analysis of the fMRI data

On the first level, regressors were modeled for each of the eight experimental conditions (i.e. the four morphing levels of male and female voices) for each subject and subsequently entered into the second level (data available from the Dryad Digital Repository: http://doi.org/10.5061/dryad.48tj0). Three different flexible factorial designs were calculated for the group analyses applying a general linear mixed-effects model (GLM) approach. Participants were entered as random effects and conditions as fixed effects. Movement parameter regressors and individual mean RTs were included as nuisance covariates. The first model contained 4 groups, namely men, women, and untreated and treated MtFs. However, no significant activation differences were found between the two MtF groups. To further analyze the effect of sexual orientation, we ran a GLM with the 4 groups (men, women, heterosexual MtFs and homosexual MtFs) and found no significant differences between hetero- and homosexual MtF groups in our investigated contrasts. Therefore, we pooled the data of all MtFs resulting in a GLM with 3 groups.

Based on this third model, main effects for original voices were calculated to investigate the neural correlates of voice perception in each group separately.

In order to compare our findings with previous results, contrasts were computed for interactions between the listeners' sex and the original voice gender [64] for the comparison between MtFs and the control groups: 1) [men 0 w > men 0 m] > [MtF 0 w > MtF 0 m], 2) [women 0 w > women 0 m] > [MtF 0 w > MtF 0 m], 3) [men 0 m > men 0 w] > [MtF 0 m > MtF 0 w], and 4) [women 0 m > women 0 w] > [MtF 0 m > MtF 0 w]. (Due to the subtraction method in the construction of contrasts, these interactions are arithmetically equivalent to 1) [MtF 0 m > MtF 0 w] > [men 0 m > men 0 w], 2) [MtF 0 m > MtF 0 w] > [women 0 m > women 0 w], 3) [MtF 0 w > MtF 0 m] > [men 0 w > men 0 m], and 4) [MtF 0 w > MtF 0 m] > [women 0 w > women 0 m]). To decompose the underlying origin of the effects, mean beta values and standard errors of each peak voxel were extracted in each group separately and for both voice conditions.

To determine the stimulus effects of gender morphing, male and female voices were weighted linearly ascending (0st*-3 < 2st*-1 < 4st*1 < 6st*3) and mean centered according to their morphing level. Resulting effects were compared between all sex groups.

For direct comparisons of men and women, see Text S1 in File S1 and Table S2 in File S1 for the interactions between the listeners' sex and the original voice gender, and Text S2 in File S1 and Table S3 in File S1 for the linear increase of voice morphing).

For all fMRI analyses, a Monte Carlo corrected threshold was applied using AlphaSim by Ward (2000) implemented in AFNI 2011 [79]. Assuming an uncorrected per voxel probability threshold of p = 0.001 and by entering the measurement parameters (e.g. voxel size, smoothing kernel), after 1.000 simulations a cluster size extent threshold of 20 contiguous re-sampled voxels was indicated to correct for multiple comparisons at p<0.05. Effects still significant after a more conservative multiple comparison cluster level correction implemented in SPM (single-voxel threshold of p<0.001 and cluster-level threshold of p<0.05, family-wise error (FWE) corrected for multiple comparisons across the whole brain) and after Bonferroni correction for multiple interaction post hoc tests (p<0.0125) are marked by asterisks.

## Results

### Behavioral results

Since we found no significant behavioral differences between treated and untreated MtFs in percentage of hits (male voices p = 0.396, female voices p = 0.720), RTs (male voices p = 0.572, female voices p = 0.940), d-prime (all p>0.100) and log ß (all p>0.345), both groups were pooled for further analyses.

#### Hit rates

Friedman tests indicated significant differences in the percentage of hits across all groups, voice gender and morphing conditions (x^2^(7) = 370.69, p<0.001).

Stimulus morphing decreased percentage of hits in the whole group and in all groups separately (all p<0.001) without group differences (p≤0.008).

An effect of voice gender was found in men (z = −2.47, p = 0.014) and women (z = −3.02, p = 0.003) with more correct answers for voices of the opposite sex. In contrast, MtFs revealed no such effect (z = −1.13, p = 0.258).

Directly comparing men and MtFs as well as men and women, differences were observed with MtFs and women performing better in response to male voices (men vs. MtFs: z = −2.672, p = 0.008; men vs. women: z = −3.921, p<0.001) and men in response to female voices (men vs. MtFs: z = −2.776, p = 0.006; men vs. women: z = −3.584, p<0.001). Comparing women and MtFs, differences regarding male and female voices (both z>−2.045, p<0.05) did not survive Bonferroni correction (p≤0.008).

**Figure 1:**
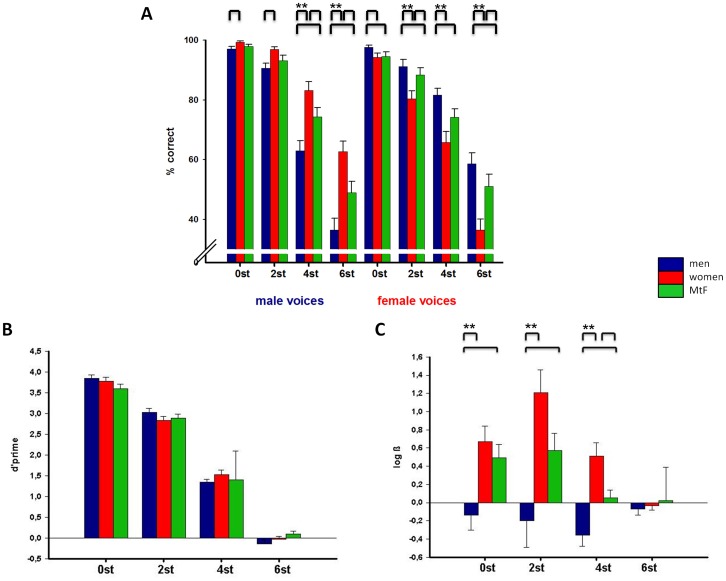
Behavioral performance. A. Performance (% hits with standard error bars) in response to male (left) and female (right) voices of the different morphing steps in semitones (st) in men (blue), women (red) and MtFs (green). Significant differences between groups are marked by bars (p<0.05) or asterisks (p≤0.002 Bonferroni corrected). B. Gender discrimination sensitivity (d-prime with standard error bars). C. Response bias (log ß with standard error bars) for each morphing step in men, women and MtFs with positive values representing bias to choose male voices and negative values representing bias to choose female voices. Significant differences are marked by bars (p<0.05) or asterisks (p≤0.002 Bonferroni corrected).

None of the comparisons of each of the 8 sub-conditions between men and MtFs as well as women and MtFs did survive Bonferroni correction (p≤0.002) though trends (p<0.05) become obvious. In contrast, comparisons between men and women revealed significant differences (see Table 2, Figure 1).

#### Discrimination and response bias

One-sample t-tests revealed discrimination ability above chance level for all conditions (p<0.001) except 6st (men: p = 0.147, women: p = 0.608, MtFs: p = 0.243). As expected, discrimination sensitivity declined as the degree of morphing increased (Figure 1). Comparisons of d' for each morphing level revealed no significant group differences (Figure 1, Table 2).

The repeated measures ANOVA for log ß revealed significant effects of morphing (F = 8.642, df = 2.1, 138.5; p<0.001) and group (F = 10.483, df = 2, 67; p<0.001) as well as an interaction of morphing and group (F = 4.932, df = 4.1, 138.5; p = 0.001).

While men revealed no significant position bias (all p≥0.293) except a trend to indicate female voices for 4st (p = 0.006), women were biased towards indicating male voices (all p≤0.004) except in the condition most morphed into the female direction (p = 0.493). Also MtFs revealed a bias towards indicating male voices for 0st (p = 0.002) and by trend for 2st (p = 0.005). Group comparisons regarding log ß reflected significant differences between men and women and by trend between men and MtFs for all morphing levels except 6st, Women and MtFs only differed by trend in the 4st condition (Figure 1, Table 2).

#### Reaction times

Friedman tests indicated significant differences in RT across all groups, voice gender and morphing conditions (x^2^(7) = 304.277, p<0.001). RT increased with morphing level (all p<0.001) with increases in RT for increased morphing. Participants as a whole group reacted faster to female compared to male voices (z = −2.848, p<0.004).

Direct group comparisons indicated women reacted faster than men in response to male voices (z = −2.894, p = 0.003) and to each morphing level (all p≤0.022). Further, women by trend reacted faster than MtFs in response to male voices (z = −2.046, p = 0.041). There was no difference between men and MtFs in any of the morphing levels (all p>0.207) or regarding the sex of the voices (both p>0. 175; Table 2).

Due to the reported group differences, we included RT as covariate into the functional imaging analyses.

### Functional imaging results

Similar to the behavioral data, we found no significant brain activation differences between treated and untreated MtFs in the processing of male vs. female original voices as well as regarding gender morphing. Therefore both groups were pooled for further analyses.

#### Effects of original voice perception

One-sample analyses revealed bilateral activation in voice-selective areas such as the bilateral superior temporal gyri and bilateral cingulate cortex (for details see Figure S1 in File S1 and Table S1 in File S1), confirming the validity of the paradigm.

#### Effects of listener sex and original voice gender

**Figure 2:**
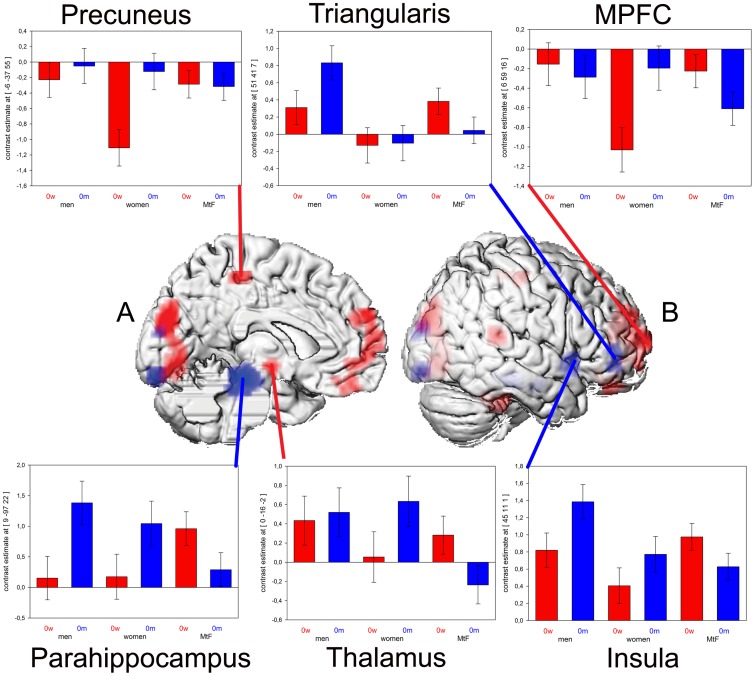
Interaction between original voice sex and group (p<0.05 Monte Carlo corrected, extent threshold  = 20 voxels). Male vs. female voices in men compared to MtFs (blue) and in women compared to MtFs (red).Parameter estimates are shown separately for male (0 m) and female (0 w) voices for men, women and MtFs A: left hemisphere, B: right hemisphere.

Interaction analyses yielded stronger activation in *men* compared to MtFs for the processing of male vs. female original voices in the right hemispheric area triangularis, insula, and cuneus, the bilateral lingual gyrus extending to the calcarine gyrus and to the parahippocampus on the left side (Table 3, Figure 2).

**Table 3:** Comparisons between groups and voice gender.

*Brain region*	*L/R*	*x*	*y*	*z*	*k*	*t*
a) men > MtFs						
Lingual gyrus extending to parahippocampal gyrus*	L	−12	−37	−11	311	4.64
Cuneus	R	9	−97	22	29	4.43
Lingual gyrus extending to calcarine gyrus	L	−9	−97	−8	50	4.02
Insula	R	45	11	1	33	3.76
Calcarine gyrus extending to lingual gyrus	R	18	−94	−5	31	3.60
Area tringularis (IFG)	R	51	41	7	26	3.59
b) women > MtFs						
Cuneus*	R	6	−91	28	348	4.78
MPFC extending to rACC*	R	6	59	16	278	4.66
MPFC extending to rACC*	L	−15	53	7	100	4.14
Cerebellum	R	12	−40	−29	49	3.92
STG	R	48	−43	16	31	3.76
Precuneus, paracentral lobe	L	−6	−37	55	35	3.73
Thalamus	-	0	−16	−2	22	3.61
Precentral gyrus	R	21	−25	58	20	3.45

Stronger activation/less deactivation in a) men compared to MtFs and b) women compared to MtFs for the processing of male vs. female original voices with no significant results for the opposite interactions ([MtF 0m > MtF 0w] > [men/women 0m > men/women 0w]; MNI coordinates, p<0.05 Monte Carlo corrected, k =  cluster extension).

*significant at SPM cluster level (p<0.0125 Bonferroni corrected).

The comparison between *women* and MtFs revealed enhanced activation for the processing of male vs. female original voices in the bilateral MPFC extending into the rostral anterior cingulate cortex (rACC), the right superior temporal gyrus (STG), precentral gyrus, cuneus, cerebellum, thalamus and the left precuneus extending into the paracentral lobe (Table 3, Figure 2).

There was no significant activation difference in a) MtFs or women compared to men in response to male vs. female voices or b) men or women compared to MtFs in response to female vs. male voices.

For a detailed description of the comparison between men and women see Junger and colleagues [64] and Text S1 in File S1 and Table S2 in File S1).

#### Effects of voice gender morphing

**Figure 3:**
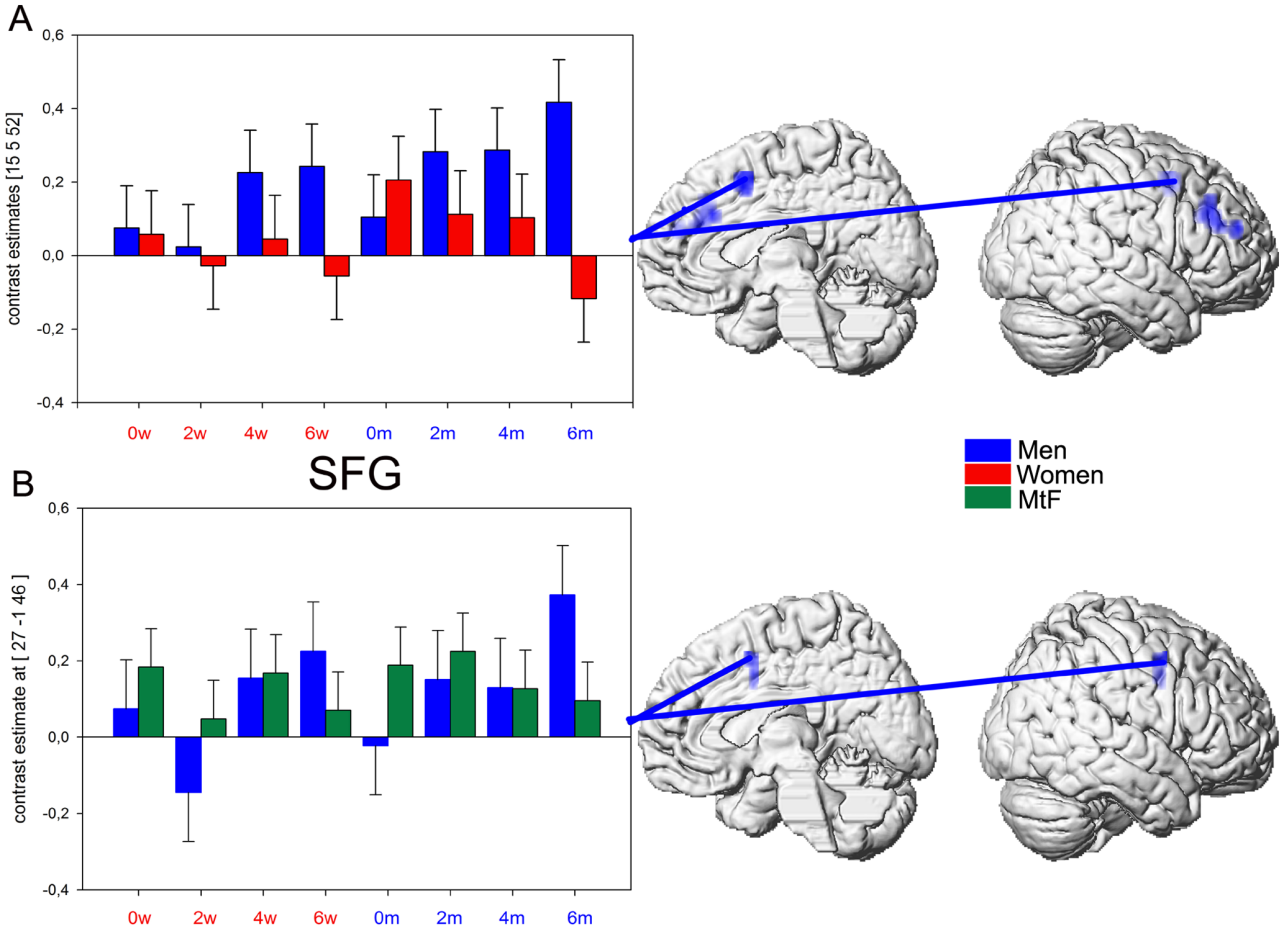
Graduate voice gender morphing. A. Contrast estimates of stronger activation in men as compared to women (blue) in the right SFG (peak voxel 15 5 52) for increasing morphing degree plotted for all 8 conditions. B. Contrast estimates of stronger activation in men as compared to MtFs (blue) in the right SFG (peak voxel 27 −1 46) for increasing morphing degree plotted for all 8 conditions (p<0.05 Monte Carlo corrected, extent threshold  = 20 voxels).

With respect to the parametric weighting of the linearly increasing morphing degree, stronger activation in the right superior frontal gyrus (SFG, MNI 27 −1 46, k = 21, t = 3.51) was found in men compared to MtFs (Figure 3). Parameter estimates reflected an overall increase in activation with higher degrees of morphing, whereas MtFs did not show such a pattern in this region. No activation difference was found for the reverse contrast (MtFs > men). There was no difference in either contrasts between women and MtFs.

## Discussion

### Hormonal level and sexual preference in MtFs

In line with the observations of Haraldsen et al. [80] and Wisnewski et al. [81], but contrary to those of Van Goozen et al. [82], [83], we found no differences between gender dysphoric individuals untreated or treated with cross-sex hormones. Differences in findings might arise from the usage of different paradigms, such as more complex visuospatial and verbal fluency tasks, known to robustly reflect gender differences on a behavioral level. However, Van Goozen et al. [84] also did not find a effect of treatment when comparing MtFs and FtMs tested prior to and 14 weeks after cross-sex hormonal treatment. The notion of possible task effects is further corroborated by the study of Miles et al. [85], who found hormone effects in a paired association learning task, but not in verbal memory and other cognitive tasks [86]. Overall, hormonal effects seem to be rather subtle both in behavioral and neurofunctional terms.

Although there is evidence from vaginal responses to visual erotic stimuli indicating differences between homo- and heterosexual MtFs [87], [88], we did not find differences in brain activation between these groups in our sample. This is possibly due to the different physiological measure and stimuli used. Further, structural brain-imaging data revealed subtle differences in the bed nucleus of the stria terminalis when comparing heterosexual men with MtFs, but not in the comparison of hetero- and homosexual men [25]. According to that, our resulting pattern measured in a sample with mixed sexual orientation is in line with various functional studies exploring different biologically relevant domains in heterosexual [89], homosexual [27], [30], [31], [37], [90] and mixed [91] gender dysphoric samples in comparison to heterosexual men and women as well as in samples with no information on sexual orientation [28], [36], [39]. This might indicate that differences between individuals with gender dysphoria and their biological sex are more pronounced than differences related to their sexual orientation. However, small sub-group sample sizes might have disguised group differences based on sexual orientation within our group of individuals with gender dysphoria.

### Differences in voice gender processing

In contrast to men and women, who showed a significantly better performance in response to voices of the opposite sex [64], [92], [93], MtFs displayed similar performance accuracy for male and female voices. Moreover, while men and women differed regarding RTs, MtFs exhibited an intermediate position with no significant differences compared to men and women. In line with the latter observation, Cohen-Kettenis and colleagues [94] proposed an intermediate position for MtFs and FtMs, placing both groups between men and women, in spatial cognition and verbal memory tasks; i.e. the respective response patterns of the groups reflected incongruence with their biological sex. Similar to these behavioural results, brain activation in MtFs differed from the other two groups when listening to male (as compared to female) voices, supporting the notion of an intermediate position between men and women.

#### Activation differences between men and MtFs

MtFs showed less activation in the parahippocampal gyrus, IFG and insula than men when contrasting male and female voices. The parahippocampus is associated with emotional auditory processing [95], related to automatic matching of incoming meaningful sounds to stored representations [96] and was found to be less active in good compared to weak learners in an auditory memory task [97]. The insula is involved in paralinguistic information processing, such as vocal identity [98] and in the creation of an acoustic “mean voice” representation. [99]. Increased IFG activation in response to a voice discrimination task has been related to increased processing demand [100], [101] and, accordingly, indicates augmented processing demands in the identification of male vs. female voices in men compared to MtFs. In line with this, the smaller activation differences in MtFs in response to male as compared to female voices may reflect a good stored representation of both male and female voices leading to a similar demand when comparing them to learned “mean voice” representations. Due to the fact that MtFs grew up with male voices and trained themselves to sound more feminine, they possess extensive experience and expertise with respect to the voices of both genders.

Activation in the IFG triangularis in particular correlated negatively with implicitly perceived vocal attractiveness [102]. Moreover, MtFs exhibited less activation than men in the lingual and calcarine gyri when processing male as compared to female voices. Given that it was an auditory task, the observed activation differences in the visual system seem surprising. However, there is evidence of activation in these areas during auditory word and pseudoword processing [103] and passive speech listening [104]. This indicates the involvement of other primary sensory cortices in auditory word processing besides the auditory cortex. In line with von Kriegstein and colleagues [59], who reported that activation in the cuneus, the lingual and calcarine gyri results from attention to the verbal content of an auditory presented sentence, we propose that activation in these regions reflects that differences were based on a differential focus of attentional processes on the semantic content rather than voice characteristics. Accordingly, this difference between male and female voice processing seems to be more pronounced in men than in MtFs.

In light of a mating-related opposite-sex effect in voice gender perception in men [64], the stronger activity in all mentioned areas (in men compared to MtFs) may reflect men's relative indifference regarding male voices.

Thus, neuronal differences between men and MtFs may be related to both mating behavior and voice gender expertise, the former resulting in less attention to masculine voices in men and the latter resulting in generally decreased activation in MtFs compared to men and women (see below). In sum, our observation provides evidence for distinct cerebral activation patterns in MtFs different from their biological sex.

#### Activation differences between women and MtFs

During the evaluation of male as compared to female voices, women revealed stronger activation than MtFs in the right STG, which is linked to affective and identity information processing inherent in vocal stimuli [56]. Moreover, voice recognition accuracy was correlated with activation in the right superior temporal area [105]. Since we found no differences in accuracy between women and MtFs, decreased activation in MtFs might suggest that they need less effort to achieve levels of performance similar to women. This might be due to the fact that MtFs are more attuned to issues related to voice gender perception in everyday life.

Women also revealed less deactivation in the precuneus. Gur and colleagues [106] demonstrated increased deactivation when contrasting target detection with novelty detection, explaining it in terms of greater attentional demands. Thus, this might indicate women pay more attention to male voices than MtFs, while MtFs revealed similar responses to male and female voices. This was also reflected in similar accuracy and RT found in MtFs for both stimulus types. The anterior region of the precuneus is known to be involved in self-centered mental imagery. Interestingly, women reveal stronger connectivity than men between the precuneus and the thalamus [107] as well as between the medial dorsal-anterior precuneus and the ACC during attentional processes [108]. This fits nicely to our observation of increased activation in all mentioned areas connected to the precuneus in women.

The ACC, finally, is linked to reward anticipation [109] and the reflection on subjective preferences [110]. In both men and women, it revealed enhanced activation in response to opposite-sex stimuli, which were construed as having greater salience [111]. Similarly, we could show its increased down-regulation in response to female voices in women and in response to male voices in men [64] with MtFs revealing activation patterns more similar to the male control sample.

Thus, parts of the reported behavioral and brain activation patterns underline an exceptional position of MtFs with qualitative differences from both men and women.

### Differences in gradual voice gender morphing

In line with Cohan and Forget [112], who could show that women and hormonally treated MtFs performed similarly in two auditory tasks, we found a response bias in MtFs more similar to their aspired gender than to their biological sex, i.e. a tendency towards indicating male voices, at least for 0st and 2st voices. Further, in the direct group comparison, MtFs as well as women performed better than men in response to male voices underlining a certain overlap with the aspired gender.

As reported before [64], we found no sex-dependent activation patterns in typical voice-selective auditory areas in response to increased morphing. Instead, we observed increased activation in the SFG in men compared to women and to MtFs. Stronger activation in the SFG has been associated with greater top-down control or cognitive effort to fulfil tasks in men compared to women [113], [114]. Men seem to require greater attentional resources and cognitive control in order to discriminate voices with increasing difficulty in contrast to women and MtFs.

Concerning increased voice gender morphing, MtFs resemble women more closely than men on both behavioral and neuronal levels. This observation is in line with other fMRI studies which found a more female-like activation pattern in MtFs [39] [89].

## Conclusions

We found sex-specific brain networks in men, women and MtFs when identifying gender by means of vocal sounds [64]. It seems that sex differences in voice gender perception are reflected in a widespread network involved in auditory, attentional, emotional and retrieval processes.

In contrast to men and women, MtFs showed no opposite-sex performance effect in voice perception. However, in direct comparison MtFs and women performed better in response to male voices and men when evaluating female voices. Further, there was a difference in RT between men and women, but none between MtFs and the two other groups. Both performance measures suggest a distinction between MtFs and men as well as women.

In line with the behavioral results, MtFs showed differences (compared to men and women) in neuronal response patterns with respect to male vs. female voices. Presumably, a different strategy is used in MtFs' voice gender identification due to early processing differences. They also might more intensively examine their own and aspired vocal characteristics during gender alignment, resulting in a certain expertise. In this sense, attentional differences due to automatized processing could lead to less brain activation in MtFs.

By morphing original voices into an ambiguous gender range, we have shown additional sex differences in terms of increased activation in prefrontal areas related to higher cognitive task demands in men compared to MtFs and women, as well as a more female-like response bias in MtFs.

In sum, our data support gender dysphoric individuals' lack of identification with their biological sex. Brain activation patterns of MtFs differed from those of men and, partly, also from those of women. Thus, differences between MtFs, men and women in voice gender processing reflect a qualitative difference in behavioral and neuronal processing that cannot be easily located on a linear continuum between men and women.

Further research is necessary to replicate our results in other tasks and samples of FtMs. Facilitating the removal of the stigma associated with gender dysphoria and providing access to care to individuals experiencing severe distress due to gender nonconformity should be considered the primary goal of research in this area.

## Supporting Information

File S1 **This file contains supporting information, including Figure S1, Text S1, Text S2, and Table S1–Table S7.** Figure S1, Brain activation in men, women and MtFs (from top to bottom) for original voices (p<0.05 Monte Carlo corrected, extent threshold  = 20 voxels) revealing activation in typical voice-related areas including the bilateral superior temporal gyri. Table S1, Brain activation in men, women and MtFs for original voices (p<0.05 Monte Carlo corrected, extent threshold  = 20 voxels). Text S1, As described in Junger and colleagues (2013), men revealed stronger activation compared to women for the processing of female vs. male original voices mainly in prefrontal areas but also in the left middle temporal gyrus (MTG). Table S2, Stronger activation/less deactivation in men compared to women for the processing of female vs. male original voices ([men 0 w > men 0 m] > [women 0 w > women 0 m]) with no significant results for the opposite contrast ([women 0 w > women 0 m] > [men 0 w > men 0 m]); (MNI coordinates, p<0.05 Monte Carlo corrected, k =  cluster extension). Text S2, As described in Junger and colleagues (2013) analyzing the parametric weighting of the linearly increasing morphing degree yielded stronger activation in right superior and middle frontal gyri in men compared to women (Table S3) with increased activation with increasing morphing degree only in men. Table S3, Activation peaks (MNI coordinates) and cluster extension (k) for a linear increase of voice morphing regarding gender identity for men contrasted to women; p<.05 Monte Carlo corrected (with no significant results for the opposite contrast). Table S4, Data points used to determine averages and summary statistics in Table 1. Table S5, Data points used to determine averages and summary statistics in Table 2 for correct responses (hits) in response to male and female voices of the different morphing steps (0, 2, 4, 6 semitones (st)). Table S6, Data points used to determine averages and summary statistics in Table 2 for reaction times (RT; in milliseconds) in response to male and female voices of the different morphing steps (0, 2, 4, 6 semitones (st)). Table S7, Data points used to determine averages and summary statistics in Table 2 for discrimination sensitivity (d-prime) and answering bias (log ß) in response to male and female voices of the different morphing steps (0, 2, 4, 6 semitones).
(DOC)
