# Supplementary material for: More than Just Two Sexes: The Neural Correlates of Voice Gender Perception in Gender Dysphoria
Source: PLoS One. 2014 Nov 6;9(11):e111672. doi: 10.1371/journal.pone.0111672 (PMC4222943; doi:10.1371/journal.pone.0111672)
Supplement: File S1 — This file contains supporting information, including Figure S1, Text S1, Text S2, and Table S1–Table S7. Figure S1, Brain activation in men, women and MtFs (from top to bottom) for original voices (p<0.05 Monte Carlo corrected, extent threshold = 20 voxels) revealing activation in typical voice-related areas including the bilateral superior temporal gyri. Table S1, Brain activation in men, women and MtFs for original voices (p<0.05 Monte Carlo corrected, extent threshold = 20 voxels). Text S1, As described in Junger and colleagues (2013), men revealed stronger activation compared to women for the processing of female vs. male original voices mainly in prefrontal areas but also in the left middle temporal gyrus (MTG). Table S2, Stronger activation/less deactivation in men compared to women for the processing of female vs. male original voices ([men 0 w > men 0 m] > [women 0 w > women 0 m]) with no significant results for the opposite contrast ([women 0 w > women 0 m] > [men 0 w > men 0 m]); (MNI coordinates, p<0.05 Monte Carlo corrected, k = cluster extension). Text S2, As described in Junger and colleagues (2013) analyzing the parametric weighting of the linearly increasing morphing degree yielded stronger activation in right superior and middle frontal gyri in men compared to women (Table S3) with increased activation with increasing morphing degree only in men. Table S3, Activation peaks (MNI coordinates) and cluster extension (k) for a linear increase of voice morphing regarding gender identity for men contrasted to women; p<.05 Monte Carlo corrected (with no significant results for the opposite contrast). Table S4, Data points used to determine averages and summary statistics in Table 1. Table S5, Data points used to determine averages and summary statistics in Table 2 for correct responses (hits) in response to male and female voices of the different morphing steps (0, 2, 4, 6 semitones (st)). Table S6, Data points used to determine averages and summary statist [file pone.0111672.s001.doc]

**Supplementary information:**

**Figure S1:**

**
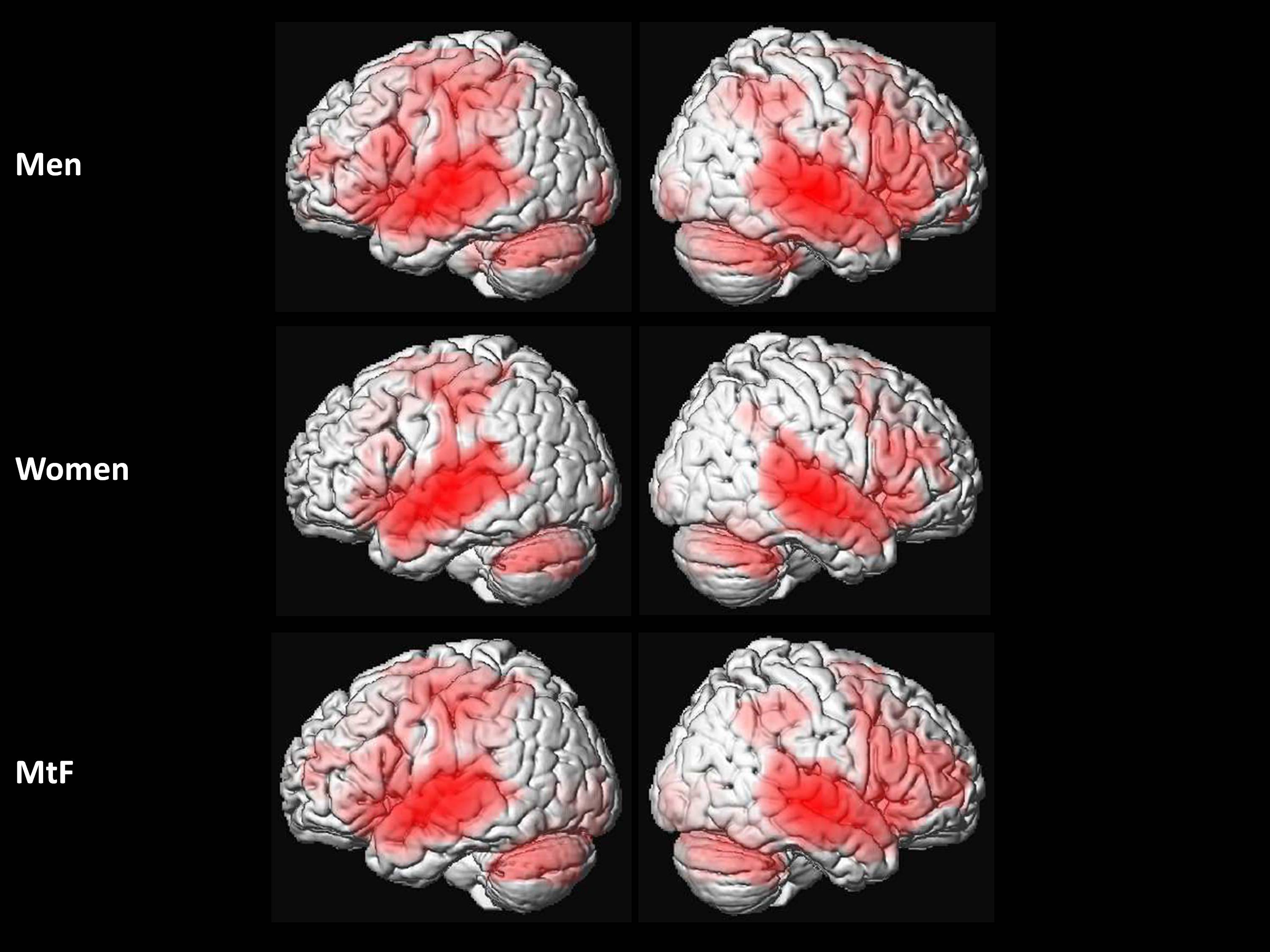
**

One sample analyses. Brain activation in men, women and MtFs (from top to bottom) for original voices (p<0.05 Monte Carlo corrected, extent threshold = 20 voxels) revealing activation in typical voice-related areas including the bilateral superior temporal gyri.

**Table S1**: Brain activation in men, women and MtFs for original voices (p<0.05 Monte Carlo corrected, extent threshold = 20 voxels)

| ***Brain region*** | ***Hemisphere*** | ***x*** | ***y*** | ***z*** | ***k*** | ***t*** |
| --- | --- | --- | --- | --- | --- | --- |
| ***Men*** |  |  |  |  |  |  |
| Superior temporal gyrus | R | 60 | -16 | 1 | 18873 | 27.03 |
| Superior temporal gyrus | L | -63 | -22 | 7 | - | 23.92 |
| Middle temporal gyrus | L | -63 | -16 | -2 |  | 23.71 |
| Precuneus | R | 9 | -67 | 43 | 136 | 5.47 |
| Cingulate region | R | 3 | -28 | 25 | 118 | 5.06 |
| Orbitofrontal gyrus | R | 24 | 56 | -11 | 33 | 4.64 |
| ***Women*** |  |  |  |  |  |  |
| Superior temporal gyrus | L | -63 | -16 | 1 | 10850 | 22.86 |
| Superior temporal gyrus | R | 63 | -16 | 1 | - | 20.96 |
| Middle temporal gyrus | L | -63 | -34 | 4 | - | 15.92 |
| Inferior parietal gyrus | R | 36 | -52 | 40 | 100 | 4.97 |
| Cingulate cortex | L | 0 | -28 | 25 | 109 | 4.97 |
| Supplementary motor area | R | 3 | 17 | 46 | 417 | 4.87 |
| Supplementary motor area | L | -6 | -1 | 52 | - | 4.80 |
| Dorsomedial prefrontal cortex | L | -6 | 17 | 40 | - | 4.71 |
| Precentral gyrus | L | -60 | 8 | 28 | 24 | 4.36 |
| Supplementary motor area | R | 6 | 8 | 70 | 51 | 4.30 |
| Supplementary motor area | L | -6 | -1 | 73 | - | 4.09 |
| Middle occipital gyrus | L | -12 | -106 | 1 | 35 | 4.15 |
| Calcarine gyrus | L | -3 | -103 | 1 | - | 3.52 |
| ***MtFs*** |  |  |  |  |  |  |
| Superior temporal gyrus | R | 63 | -13 | -5 | 18083 | 26.78 |
| Superior temporal gyrus | L | -63 | -16 | 1 | - | 25.20 |
| Middle temporal gyrus | L | -66 | -28 | 1 | - | 22.04 |

**Text S1**: As described in Junger and colleagues (2013), *men* revealed stronger activation compared to women for the processing of female vs. male original voices mainly in prefrontal areas but also in the left middle temporal gyrus (MTG). The corresponding data resulting from the actual GLM (including three groups) are presented in Table S2 (activation in the MTG did not reach significance, here).

**Table S2**: Stronger activation/less deactivation in men compared to women for the processing of female vs. male original voices ([men 0w > men 0m] > [women 0w > women 0m]) with no significant results for the opposite contrast ([women 0w > women 0m] > [men 0w > men 0m]); (MNI coordinates, p<0.05 Monte Carlo corrected, k = cluster extension).

| ***Brain region*** | ***L/R*** | ***x*** | ***y*** | ***z*** | ***k*** | ***t*** |
| --- | --- | --- | --- | --- | --- | --- |
| Medial prefrontal cortex | R | 15 | 56 | 16 | 120 | 4.48 |
| Medial orbitofrontal cortex | L | -12 | 35 | -14 | 27 | 4.06 |

**Text S2**: As described in Junger and colleagues (2013) analyzing the parametric weighting of the linearly increasing morphing degree yielded stronger activation in right superior and middle frontal gyri in men compared to women (Table S3) with increased activation with increasing morphing degree only in men.

**Table S3:** Activation peaks (MNI coordinates) and cluster extension (k) for a linear increase of voice morphing regarding gender identity for men contrasted to women; p<.05 Monte Carlo corrected (with no significant results for the opposite contrast)

| ***Brain region*** | ***L/R*** | ***x*** | ***y*** | ***z*** | ***k*** | ***t*** |
| --- | --- | --- | --- | --- | --- | --- |
| Superior frontal gyrus | R | 15 | 5 | 52 | 47 | 4.66 |
| Middle frontal gyrus | R | 30 | 29 | 31 | 88 | 4.04 |

**Table S4:** Data points used to determine averages and summary statistics in Table 1. Participants: Cm=Control male, Cf=control female, MtF=male-to-female gender dysphoric individual; groups: 1= men, 2=women, 3=untreated MtFs, 4=treated MtFs; education (in years), sexual orientation: 1=heterosexual, 2=homosexual, 3=bisexual.

| Participant | Group | Age | Education | IQ | Sexual Orientation | 17-ß-Estradiol | FSH | LH | Progesterone | Prolactin | SHBG | Testosterone |
| --- | --- | --- | --- | --- | --- | --- | --- | --- | --- | --- | --- | --- |
| Cm01 | 1,00 | 32 | 18 | 118,00 | 1,00 | 55,70 | 5,80 | 8,30 | 2,20 | 218,00 | 27,70 | 49,60 |
| Cm02 | 1,00 | 32 | 18 | 130,00 | 1,00 | 73,00 | 5,80 | 5,80 | 1,20 | 70,00 | 12,90 | 57,80 |
| Cm03 | 1,00 | 42 | 15 | 94,00 | 1,00 | 48,90 | 16,00 | 7,60 | 2,00 | 244,00 | 21,40 | 26,20 |
| Cm04 | 1,00 | 30 | 16 | 124,00 | 1,00 | 64,10 | 1,80 | 6,10 | 3,00 | 117,00 | 15,00 | 38,00 |
| Cm05 | 1,00 | 42 | 9 | 118,00 | 1,00 | 47,50 | 4,80 | 5,00 | ,80 | 258,00 | 32,50 | 31,10 |
| Cm06 | 1,00 | 25 | 16 | 118,00 | 1,00 | 82,10 | 1,40 | 3,20 | 2,40 | 143,00 | 29,30 | 35,20 |
| Cm07 | 1,00 | 36 | 18 | 145,00 | 1,00 | 51,00 | 4,70 | 6,30 | 1,40 | 85,00 | 37,40 | 75,60 |
| Cm08 | 1,00 | 27 | 16 | 95,00 | 1,00 | 144,00 | 4,10 | 8,10 | 4,60 | 162,00 | 31,50 | 67,20 |
| Cm09 | 1,00 | 28 | 16 | 100,00 | 1,00 | 82,20 | 6,20 | 8,00 | 2,70 | 111,00 | 23,40 | 37,60 |
| Cm10 | 1,00 | 46 | 10 | 101,00 | 1,00 | 82,30 | 25,70 | 9,10 | 1,00 | 163,00 | 30,00 | 27,60 |
| Cm11 | 1,00 | 32 | 16 | 118,00 | 1,00 | 90,60 | 3,80 | 3,10 | 1,90 | 196,00 | 11,80 | 22,20 |
| Cm12 | 1,00 | 19 | 13 | 107,00 | 1,00 | 141,00 | 2,00 | 4,50 | 2,80 | 163,00 | 26,80 | 37,10 |
| Cm13 | 1,00 | 21 | 16 | 104,00 | 1,00 | 70,90 | 2,10 | 6,10 | 4,00 | 161,00 | 21,00 | 37,80 |
| Cm14 | 1,00 | 52 | 18 | 124,00 | 9,00 | 117,00 | 2,50 | 6,70 | 2,20 | 130,00 | 58,20 | 48,20 |
| Cm15 | 1,00 | 23 | 18 | 94,00 | 1,00 | 116,00 | 2,40 | 3,30 | 1,70 | 221,00 | 22,20 | 28,50 |
| Cm17 | 1,00 | 23 | 13 | 101,00 | 2,00 | 122,00 | 1,00 | 4,10 | 2,90 | 96,00 | 42,60 | 43,00 |
| Cm18 | 1,00 | 50 | 18 | 136,00 | 1,00 | - | - | - | - | - | - | - |
| Cm19 | 1,00 | 21 | 13 | 97,00 | 1,00 | 54,80 | 3,00 | 5,80 | 1,70 | 254,00 | 31,70 | 39,20 |
| Cm20 | 1,00 | 43 | 10 | 118,00 | 1,00 | - | - | - | - | - | - | - |
| Cm21 | 1,00 | 23 | 13 | 107,00 | 1,00 | 152,00 | 3,20 | 2,60 | 3,20 | 199,00 | 26,40 | 30,90 |
| Cf01 | 2,00 | 25 | 16 | 101,00 | 1,00 | 18,40 | ,50 | ,10 | - | 207,00 | - | ,30 |
| Cf02 | 2,00 | 25 | 16 | 130,00 | 1,00 | 18,40 | 1,00 | ,10 | 1,00 | 142,00 | 180,00 | 5,90 |
| Cf03 | 2,00 | 30 | 18 | 104,00 | 1,00 | 321,00 | 5,50 | 13,00 | 1,80 | 146,00 | 87,80 | 3,80 |
| Cf04 | 2,00 | 23 | 18 | 136,00 | 1,00 | 41,40 | ,20 | ,10 | 3,10 | 263,00 | 200,00 | 2,80 |
| Cf05 | 2,00 | 27 | 16 | 95,00 | 1,00 | 18,40 | 2,70 | ,70 | 1,50 | 224,00 | 176,50 | 2,00 |
| Cf06 | 2,00 | 27 | 16 | 104,00 | 9,00 | 37,10 | 4,70 | 9,90 | ,80 | 150,00 | 200,00 | 2,00 |
| Cf07 | 2,00 | 25 | 18 | 97,00 | 1,00 | 46,10 | 1,60 | 1,30 | 1,40 | 296,00 | 195,50 | 1,30 |
| Cf08 | 2,00 | 23 | 16 | 101,00 | 1,00 | 54,20 | 6,60 | 4,50 | 1,00 | 187,00 | 200,00 | 1,70 |
| Cf09 | 2,00 | 23 | 18 | 130,00 | 1,00 | 134,00 | 4,90 | 3,80 | 1,30 | 214,00 | 38,50 | 6,90 |
| Cf10 | 2,00 | 27 | 16 | 104,00 | 1,00 | 44,10 | ,80 | ,30 | 1,50 | 183,00 | 182,90 | 3,10 |
| Cf11 | 2,00 | 52 | 10 | 107,00 | 1,00 | 409,00 | 47,90 | 49,10 | 1,90 | 125,00 | 62,50 | 5,90 |
| Cf13 | 2,00 | 49 | 18 | 130,00 | 1,00 | 379,00 | 5,60 | 2,90 | 28,20 | 292,00 | 84,00 | 2,40 |
| Cf14 | 2,00 | 38 | 18 | 124,00 | 1,00 | 136,00 | 7,50 | 5,50 | 2,20 | 170,00 | 27,90 | 6,60 |
| Cf15 | 2,00 | 54 | 16 | 145,00 | 1,00 | 32,20 | 95,50 | 46,90 | ,70 | 192,00 | 167,80 | 2,10 |
| Cf16 | 2,00 | 20 | 10 | 93,00 | 1,00 | 18,40 | ,10 | ,10 | ,40 | 193,00 | 200,00 | 1,40 |
| Cf17 | 2,00 | 52 | 12 | 124,00 | 1,00 | 180,00 | 54,00 | 28,70 | 1,50 | 131,00 | 88,20 | 6,60 |
| Cf18 | 2,00 | 19 | 10 | 95,00 | 1,00 | 161,00 | 6,70 | 18,50 | 3,40 | 221,00 | 48,40 | 8,30 |
| Cf19 | 2,00 | 43 | 13 | 112,00 | 1,00 | 189,00 | 7,30 | 9,60 | ,30 | 276,00 | 62,00 | 3,10 |
| Cf20 | 2,00 | 48 | 9 | 100,00 | 1,00 | 347,00 | 2,30 | ,90 | 28,30 | 101,00 | 99,00 | 4,90 |
| MtF01 | 3,00 | 52 | 18 | 124,00 | 2,00 | 31,00 | 5,30 | 3,00 | 1,50 | 114,00 | 32,30 | 34,10 |
| MtF02 | 3,00 | 40 | 18 | 104,00 | 1,00 | 31,30 | 5,80 | 7,00 | 1,80 | 238,00 | 42,20 | 53,30 |
| MtF03 | 4,00 | 40 | 18 | 104,00 | 2,00 | 219,00 | 16,20 | 6,70 | 3,40 | 154,00 | 56,70 | 23,70 |
| MtF04 | 3,00 | 23 | 12 | 118,00 | 1,00 | 75,90 | 4,20 | 6,10 | 3,00 | 104,00 | 19,80 | 40,50 |
| MtF05 | 4,00 | 24 | 16 | 112,00 | 1,00 | 694,00 | ,10 | ,10 | 1,00 | 961,00 | 27,90 | 2,00 |
| MtF06 | 3,00 | 51 | 18 | 104,00 | 2,00 | 93,40 | 5,60 | 8,20 | 1,70 | 309,00 | 36,30 | 31,40 |
| MtF07 | 3,00 | 36 | 12 | 107,00 | 3,00 | 95,40 | 3,50 | 3,60 | 2,40 | 157,00 | 67,10 | 31,00 |
| MtF08 | 3,00 | 19 | 12 | 101,00 | 1,00 | 141,00 | 2,10 | 2,00 | 3,50 | 191,00 | 10,30 | 32,70 |
| MtF09 | 4,00 | 20 | 16 | 107,00 | 2,00 | 878,00 | ,50 | ,50 | 1,50 | 1037,00 | 28,10 | 2,10 |
| MtF10 | 3,00 | 24 | 12 | 118,00 | 1,00 | 118,00 | 4,70 | 2,50 | 1,80 | 169,00 | 19,30 | 24,50 |
| MtF11 | 4,00 | 43 | 10 | 104,00 | 3,00 | 539,00 | 1,00 | ,10 | 1,20 | 521,00 | 115,90 | 2,70 |
| MtF12 | 4,00 | 54 | 9 | 104,00 | 2,00 | 245,00 | ,20 | ,10 | ,70 | 331,00 | 64,40 | 1,60 |
| MtF13 | 3,00 | 38 | 10 | 104,00 | 1,00 | 35,70 | 8,90 | 7,20 | ,90 | 102,00 | 31,80 | 30,70 |
| MtF14 | 4,00 | 33 | 18 | 104,00 | 2,00 | 1020,00 | 21,30 | 13,00 | 1,70 | 178,00 | 58,10 | 1,60 |
| MtF15 | 4,00 | 32 | 18 | 107,00 | 2,00 | 92,40 | 31,80 | 36,70 | 1,10 | 180,00 | 43,90 | 1,30 |
| MtF16 | 3,00 | 32 | 12 | 118,00 | 3,00 | 101,00 | 10,80 | 7,40 | 3,20 | 85,00 | 35,80 | 51,00 |
| MtF17 | 3,00 | 21 | 16 | 107,00 | 1,00 | 146,00 | 3,80 | 7,00 | 2,00 | 120,00 | 28,30 | 43,70 |
| MtF18 | 4,00 | 32 | 9 | 95,00 | 1,00 | 412,00 | ,10 | ,10 | 1,50 | 210,00 | 77,60 | 4,20 |
| MtF19 | 4,00 | 19 | 12 | 97,00 | 1,00 | 498,00 | ,20 | ,10 | 2,40 | 471,00 | 58,40 | 6,30 |
| MtF20 | 3,00 | 19 | 12 | 94,00 | 1,00 | 216,00 | ,80 | 3,40 | 2,20 | 211,00 | 38,20 | 36,80 |
| MtF23 | 4,00 | 26 | 14 | 100,00 | 1,00 | 2010,00 | ,10 | ,10 | 2,50 | 2112,00 | 82,50 | 3,10 |
| MtF24 | 3,00 | 47 | 12 | 112,00 | 2,00 | 101,00 | 7,60 | 13,00 | 1,50 | 125,00 | 29,50 | 13,50 |
| MtF25 | 4,00 | 26 | 16 | 112,00 | 1,00 | - | - | - | - | - | - | - |
| MtF26 | 4,00 | 18 | 12 | 118,00 | 1,00 | 1050,00 | ,10 | ,10 | 1,00 | 551,00 | 273,30 | 1,40 |
| MtF27 | 4,00 | 20 | 13 | 95,00 | 1,00 | 304,00 | ,10 | ,10 | 1,90 | 315,00 | 60,30 | 3,10 |
| MtF28 | 4,00 | 48 | 14 | 97,00 | 3,00 | 302,00 | 11,70 | 4,20 | 1,00 | 127,00 | 162,60 | 5,20 |
| MtF29 | 3,00 | 51 | 18 | 118,00 | 2,00 | 89,90 | 4,70 | 3,30 | 2,20 | 136,00 | 23,30 | 35,70 |
| MtF30 | 3,00 | 61 | 18 | 145,00 | 2,00 | 18,40 | ,50 | ,50 | ,30 | 248,00 | 176,30 | 1,00 |
| MtF31 | 3,00 | 47 | 18 | 136,00 | 2,00 | 63,70 | 3,30 | 4,20 | ,70 | 111,00 | - | - |
| MtF32 | 4,00 | 26 | 12 | 100,00 | 1,00 | 12600,00 | ,10 | ,10 | 2,80 | 1117,00 | 215,20 | - |
| MtF33 | 4,00 | 22 | 14 | 112,00 | 1,00 | 138,00 | ,10 | ,10 | 2,50 | 307,00 | 42,90 | - |
| MtF34 | 3,00 | 21 | 14 | 100,00 | 2,00 | 109,00 | 2,30 | 6,40 | 4,20 | 228,00 | 13,70 | - |

**Table S5:** Data points used to determine averages and summary statistics in Table 2 for correct responses (hits) in response to male and female voices of the different morphing steps (0, 2, 4, 6 semitones (st)). Participants: Cm=Control male, Cf=control female, MtF=male-to-female gender dysphoric individual.

| Participant | Hits 0st male | Hits 0st female | Hits 2st male | Hits 2st female | Hits 4st male | Hits 4st female | Hits 6st male | Hits 6st female |
| --- | --- | --- | --- | --- | --- | --- | --- | --- |
| Cm01 | 96,67 | 100,00 | 80,00 | 96,67 | 43,33 | 93,33 | 16,67 | 86,67 |
| Cm02 | 100,00 | 93,33 | 96,67 | 80,00 | 90,00 | 73,33 | 70,00 | 46,67 |
| Cm03 | 100,00 | 100,00 | 96,67 | 93,33 | 73,33 | 83,33 | 46,67 | 70,00 |
| Cm04 | 96,67 | 100,00 | 83,33 | 100,00 | 50,00 | 93,33 | 20,00 | 73,33 |
| Cm05 | 96,67 | 100,00 | 86,67 | 100,00 | 46,67 | 80,00 | 23,33 | 66,67 |
| Cm06 | 100,00 | 100,00 | 100,00 | 90,00 | 73,33 | 83,33 | 46,67 | 53,33 |
| Cm07 | 96,67 | 100,00 | 86,67 | 100,00 | 60,00 | 93,33 | 40,00 | 76,67 |
| Cm08 | 100,00 | 100,00 | 96,67 | 100,00 | 63,33 | 80,00 | 30,00 | 53,33 |
| Cm09 | 93,33 | 96,67 | 93,33 | 83,33 | 70,00 | 73,33 | 53,33 | 53,33 |
| Cm10 | 90,00 | 96,67 | 96,67 | 70,00 | 83,33 | 70,00 | 56,67 | 36,67 |
| Cm11 | 100,00 | 96,67 | 96,67 | 100,00 | 60,00 | 80,00 | 36,67 | 70,00 |
| Cm12 | 100,00 | 100,00 | 93,33 | 86,67 | 66,67 | 86,67 | 33,33 | 70,00 |
| Cm13 | 100,00 | 100,00 | 83,33 | 93,33 | 53,33 | 90,00 | 36,67 | 56,67 |
| Cm14 | 100,00 | 90,00 | 100,00 | 60,00 | 90,00 | 53,33 | 70,00 | 16,67 |
| Cm15 | 90,00 | 96,67 | 83,33 | 100,00 | 43,33 | 83,33 | 13,33 | 53,33 |
| Cm17 | 96,67 | 96,67 | 90,00 | 90,00 | 70,00 | 73,33 | 26,67 | 46,67 |
| Cm18 | 93,33 | 96,67 | 76,67 | 93,33 | 40,00 | 83,33 | 6,67 | 66,67 |
| Cm19 | 100,00 | 96,67 | 96,67 | 93,33 | 73,33 | 86,67 | 36,67 | 40,00 |
| Cm20 | 100,00 | 93,33 | 80,00 | 100,00 | 56,67 | 90,00 | 30,00 | 70,00 |
| Cm21 | 90,00 | 100,00 | 96,67 | 93,33 | 53,33 | 83,33 | 36,67 | 66,67 |
| Cf01 | 100,00 | 96,67 | 90,00 | 100,00 | 70,00 | 73,33 | 46,67 | 43,33 |
| Cf02 | 100,00 | 93,33 | 96,67 | 70,00 | 90,00 | 50,00 | 80,00 | 33,33 |
| Cf03 | 100,00 | 96,67 | 96,67 | 86,67 | 76,67 | 76,67 | 50,00 | 50,00 |
| Cf04 | 96,67 | 96,67 | 96,67 | 86,67 | 96,67 | 80,00 | 70,00 | 43,33 |
| Cf05 | 100,00 | 96,67 | 96,67 | 80,00 | 96,67 | 53,33 | 76,67 | 13,33 |
| Cf06 | 100,00 | 96,67 | 100,00 | 76,67 | 93,33 | 50,00 | 73,33 | 20,00 |
| Cf07 | 93,33 | 100,00 | 90,00 | 86,67 | 90,00 | 63,33 | 63,33 | 36,67 |
| Cf08 | 100,00 | 96,67 | 100,00 | 90,00 | 90,00 | 83,33 | 73,33 | 43,33 |
| Cf09 | 100,00 | 96,67 | 96,67 | 86,67 | 66,67 | 80,00 | 43,33 | 56,67 |
| Cf10 | 100,00 | 96,67 | 96,67 | 83,33 | 73,33 | 70,00 | 56,67 | 53,33 |
| Cf11 | 100,00 | 80,00 | 100,00 | 53,33 | 93,33 | 30,00 | 83,33 | 6,67 |
| Cf13 | 100,00 | 90,00 | 100,00 | 66,67 | 86,67 | 60,00 | 73,33 | 20,00 |
| Cf14 | 100,00 | 100,00 | 86,67 | 83,33 | 50,00 | 86,67 | 36,67 | 60,00 |
| Cf15 | 100,00 | 80,00 | 100,00 | 63,33 | 96,67 | 46,67 | 86,67 | 16,67 |
| Cf16 | 96,67 | 96,67 | 100,00 | 76,67 | 86,67 | 66,67 | 53,33 | 30,00 |
| Cf17 | 100,00 | 96,67 | 100,00 | 86,67 | 90,00 | 73,33 | 56,67 | 33,33 |
| Cf18 | 100,00 | 100,00 | 93,33 | 100,00 | 66,67 | 86,67 | 33,33 | 60,00 |
| Cf19 | 100,00 | 96,67 | 100,00 | 76,67 | 80,00 | 73,33 | 66,67 | 40,00 |
| Cf20 | 100,00 | 83,33 | 100,00 | 73,33 | 86,67 | 46,67 | 66,67 | 33,33 |
| MtF01 | 96,67 | 96,67 | 100,00 | 86,67 | 83,33 | 70,00 | 50,00 | 40,00 |
| MtF02 | 100,00 | 100,00 | 100,00 | 96,67 | 86,67 | 83,33 | 60,00 | 53,33 |
| MtF03 | 96,67 | 86,67 | 100,00 | 76,67 | 80,00 | 70,00 | 53,33 | 60,00 |
| MtF04 | 96,67 | 100,00 | 93,33 | 96,67 | 73,33 | 70,00 | 43,33 | 46,67 |
| MtF05 | 100,00 | 93,33 | 96,67 | 83,33 | 83,33 | 56,67 | 73,33 | 33,33 |
| MtF06 | 100,00 | 93,33 | 90,00 | 86,67 | 76,67 | 83,33 | 40,00 | 53,33 |
| MtF07 | 100,00 | 93,33 | 96,67 | 86,67 | 90,00 | 60,00 | 73,33 | 43,33 |
| MtF08 | 100,00 | 93,33 | 100,00 | 93,33 | 83,33 | 70,00 | 53,33 | 50,00 |
| MtF09 | 100,00 | 73,33 | 100,00 | 63,33 | 100,00 | 43,33 | 86,67 | 23,33 |
| MtF10 | 100,00 | 76,67 | 96,67 | 73,33 | 86,67 | 56,67 | 70,00 | 33,33 |
| MtF11 | 93,33 | 96,67 | 80,00 | 86,67 | 63,33 | 76,67 | 43,33 | 70,00 |
| MtF12 | 96,67 | 93,33 | 93,33 | 86,67 | 73,33 | 66,67 | 46,67 | 53,33 |
| MtF13 | 93,33 | 96,67 | 80,00 | 96,67 | 56,67 | 73,33 | 26,67 | 56,67 |
| MtF14 | 100,00 | 90,00 | 100,00 | 83,33 | 90,00 | 60,00 | 66,67 | 40,00 |
| MtF15 | 96,67 | 100,00 | 83,33 | 96,67 | 43,33 | 83,33 | 13,33 | 63,33 |
| MtF16 | 100,00 | 100,00 | 90,00 | 93,33 | 60,00 | 86,67 | 30,00 | 66,67 |
| MtF17 | 96,67 | 93,33 | 76,67 | 73,33 | 66,67 | 76,67 | 43,33 | 46,67 |
| MtF18 | 100,00 | 100,00 | 100,00 | 90,00 | 63,33 | 80,00 | 33,33 | 66,67 |
| MtF19 | 96,67 | 93,33 | 80,00 | 90,00 | 73,33 | 76,67 | 30,00 | 46,67 |
| MtF20 | 100,00 | 86,67 | 100,00 | 76,67 | 90,00 | 56,67 | 66,67 | 33,33 |
| MtF23 | 90,00 | 70,00 | 80,00 | 73,33 | 83,33 | 56,67 | 53,33 | 30,00 |
| MtF24 | 100,00 | 96,67 | 100,00 | 70,00 | 76,67 | 70,00 | 70,00 | 20,00 |
| MtF25 | 100,00 | 86,67 | 93,33 | 96,67 | 70,00 | 83,33 | 50,00 | 63,33 |
| MtF26 | 100,00 | 93,33 | 96,67 | 93,33 | 80,00 | 80,00 | 63,33 | 66,67 |
| MtF27 | 100,00 | 100,00 | 96,67 | 90,00 | 83,33 | 56,67 | 66,67 | 40,00 |
| MtF28 | 100,00 | 100,00 | 100,00 | 86,67 | 73,33 | 86,67 | 53,33 | 66,67 |
| MtF29 | 96,67 | 100,00 | 90,00 | 93,33 | 56,67 | 83,33 | 40,00 | 63,33 |
| MtF30 | 93,33 | 93,33 | 96,67 | 93,33 | 63,33 | 66,67 | 30,00 | 56,67 |
| MtF31 | 100,00 | 93,33 | 90,00 | 96,67 | 63,33 | 83,33 | 43,33 | 63,33 |
| MtF32 | 100,00 | 100,00 | 96,67 | 90,00 | 90,00 | 73,33 | 76,67 | 53,33 |
| MtF33 | 100,00 | 100,00 | 100,00 | 90,00 | 90,00 | 73,33 | 76,67 | 36,67 |
| MtF34 | 93,33 | 100,00 | 90,00 | 100,00 | 76,67 | 96,67 | 43,33 | 90,00 |

**Table S6:** Data points used to determine averages and summary statistics in Table 2 for reaction times (RT; in milliseconds) in response to male and female voices of the different morphing steps (0, 2, 4, 6 semitones (st)). Participants: Cm=Control male, Cf=control female, MtF=male-to-female gender dysphoric individual.

| Participant | RT 0st male | RT 0st female | RT 2st male | RT 2st female | RT 4st male | RT 4st female | RT 6st male | RT 6st female |
| --- | --- | --- | --- | --- | --- | --- | --- | --- |
| Cm01 | 1164,88 | 1069,49 | 1272,09 | 1101,08 | 1289,79 | 1104,52 | 1250,70 | 1165,42 |
| Cm02 | 916,51 | 1039,12 | 938,89 | 1087,37 | 1074,39 | 1066,69 | 1211,44 | 1095,68 |
| Cm03 | 1435,25 | 1344,84 | 1630,47 | 1499,57 | 1926,49 | 1445,44 | 1933,69 | 1567,41 |
| Cm04 | 1098,48 | 1003,90 | 1153,74 | 1112,07 | 1440,57 | 1095,68 | 1381,17 | 1359,91 |
| Cm05 | 1247,01 | 1159,55 | 1322,04 | 1251,41 | 1677,35 | 1220,67 | 1600,77 | 1452,90 |
| Cm06 | 1215,66 | 1188,11 | 1293,85 | 1346,96 | 1520,67 | 1372,90 | 1476,72 | 1337,85 |
| Cm07 | 1429,32 | 1436,32 | 1753,54 | 1389,03 | 1938,09 | 1392,23 | 1895,08 | 1574,22 |
| Cm08 | 1095,38 | 1113,82 | 1098,31 | 1111,78 | 1257,49 | 1184,93 | 1239,28 | 1306,57 |
| Cm09 | 1549,43 | 1550,67 | 1572,38 | 1753,45 | 2026,48 | 1768,81 | 2003,50 | 1772,55 |
| Cm10 | 906,28 | 877,50 | 969,98 | 1021,31 | 1063,94 | 986,73 | 1028,14 | 992,66 |
| Cm11 | 1099,36 | 1170,62 | 1496,64 | 1368,77 | 1713,43 | 1438,96 | 1699,54 | 1534,06 |
| Cm12 | 1258,97 | 1251,07 | 1320,68 | 1326,70 | 1452,06 | 1425,78 | 1599,95 | 1463,13 |
| Cm13 | 1155,09 | 1399,62 | 1472,37 | 1495,52 | 1619,57 | 1498,96 | 1894,55 | 1394,67 |
| Cm14 | 1135,13 | 1438,70 | 1165,73 | 1425,70 | 1385,05 | 1512,79 | 1487,26 | 1394,19 |
| Cm15 | 1160,86 | 1095,29 | 1263,27 | 1246,92 | 1261,54 | 1192,27 | 1319,00 | 1229,22 |
| Cm17 | 1412,77 | 1449,03 | 1406,32 | 1866,92 | 2118,71 | 1792,35 | 1949,71 | 1873,15 |
| Cm18 | 1315,07 | 1240,39 | 1541,74 | 1248,13 | 1530,08 | 1370,20 | 1459,70 | 1571,12 |
| Cm19 | 1104,11 | 1119,77 | 1171,20 | 1174,83 | 1512,91 | 1211,64 | 1382,32 | 1250,21 |
| Cm20 | 1094,86 | 1099,71 | 1204,19 | 1127,04 | 1443,79 | 1219,50 | 1272,17 | 1224,31 |
| Cm21 | 1605,26 | 1879,74 | 1803,96 | 2088,11 | 2039,73 | 1923,86 | 2074,72 | 1901,37 |
| Cf01 | 1239,92 | 1042,59 | 1262,53 | 1086,36 | 1355,93 | 1188,97 | 1314,21 | 1248,41 |
| Cf02 | 1121,49 | 1340,45 | 1265,37 | 1394,43 | 1328,03 | 1327,89 | 1426,19 | 1311,56 |
| Cf03 | 1155,41 | 1230,88 | 1243,03 | 1197,68 | 1241,55 | 1091,38 | 1406,48 | 1336,87 |
| Cf04 | 1271,34 | 1191,44 | 1348,64 | 1346,71 | 1263,47 | 1539,42 | 1431,61 | 1381,90 |
| Cf05 | 1303,34 | 1285,88 | 1278,59 | 1489,97 | 1469,27 | 1685,60 | 1542,88 | 1673,03 |
| Cf06 | 766,64 | 876,57 | 889,57 | 929,54 | 914,90 | 971,69 | 969,75 | 956,12 |
| Cf07 | 896,38 | 866,82 | 915,21 | 966,72 | 1046,76 | 1148,35 | 1082,14 | 1035,15 |
| Cf08 | 1047,73 | 1061,28 | 1098,55 | 1005,24 | 1154,39 | 1170,73 | 1260,96 | 1113,88 |
| Cf09 | 1016,46 | 1079,46 | 1149,28 | 1140,49 | 1264,90 | 1193,60 | 1391,71 | 1185,00 |
| Cf10 | 1073,69 | 1085,55 | 1173,50 | 1170,07 | 1327,29 | 1214,06 | 1270,73 | 1277,75 |
| Cf11 | 1022,92 | 1009,43 | 1022,06 | 1186,95 | 1114,28 | 1168,60 | 1199,66 | 1083,21 |
| Cf13 | 1263,18 | 1293,24 | 1290,90 | 1787,63 | 1484,77 | 1618,84 | 1493,75 | 1398,17 |
| Cf14 | 813,00 | 742,09 | 1034,79 | 925,53 | 1234,39 | 992,39 | 1246,94 | 1094,57 |
| Cf15 | 947,41 | 1051,33 | 1005,52 | 1174,29 | 1160,18 | 1287,26 | 1329,70 | 1121,77 |
| Cf16 | 1152,60 | 1307,74 | 1327,79 | 1409,23 | 1373,97 | 1399,15 | 1550,80 | 1396,71 |
| Cf17 | 992,23 | 1040,59 | 1069,75 | 1111,95 | 1270,87 | 1130,10 | 1251,01 | 1148,20 |
| Cf18 | 1122,04 | 1089,80 | 1199,70 | 1134,53 | 1179,59 | 1187,38 | 1282,44 | 1225,28 |
| Cf19 | 1004,73 | 975,70 | 1086,44 | 1140,53 | 1233,56 | 1169,23 | 1218,36 | 1221,67 |
| Cf20 | 949,05 | 1209,98 | 1126,31 | 1404,42 | 1408,87 | 1639,26 | 1596,17 | 1465,39 |
| MtF01 | 1040,69 | 991,32 | 1099,48 | 1174,03 | 1366,78 | 1217,23 | 1415,85 | 1334,27 |
| MtF02 | 1075,24 | 1160,36 | 1175,54 | 1177,32 | 1329,34 | 1283,89 | 1603,33 | 1287,76 |
| MtF03 | 1434,01 | 1582,60 | 1508,55 | 1679,13 | 1535,95 | 1596,46 | 1932,02 | 1661,60 |
| MtF04 | 1373,16 | 1493,59 | 1730,17 | 1683,49 | 1834,92 | 1608,20 | 2171,53 | 1780,61 |
| MtF05 | 1148,09 | 1320,23 | 1177,38 | 1477,29 | 1307,78 | 1448,58 | 1419,99 | 1399,03 |
| MtF06 | 1025,95 | 970,36 | 1178,74 | 1148,53 | 1113,85 | 1113,74 | 1277,71 | 1173,21 |
| MtF07 | 1099,46 | 1200,67 | 1336,22 | 1563,82 | 1440,53 | 1324,24 | 1614,84 | 1539,45 |
| MtF08 | 1150,77 | 1345,28 | 1303,33 | 1370,87 | 1543,89 | 1504,21 | 1639,54 | 1345,20 |
| MtF09 | 988,76 | 1073,36 | 969,51 | 1160,41 | 1112,88 | 1280,51 | 1249,65 | 1240,56 |
| MtF10 | 1216,97 | 1441,90 | 1236,12 | 1375,96 | 1345,67 | 1193,55 | 1408,35 | 1388,65 |
| MtF11 | 1223,85 | 1304,99 | 1288,85 | 1458,42 | 1678,16 | 1507,01 | 1718,07 | 1489,82 |
| MtF12 | 1013,59 | 1159,98 | 1028,49 | 1174,56 | 1200,74 | 1109,94 | 1242,36 | 1173,62 |
| MtF13 | 1285,82 | 1174,45 | 1341,27 | 1428,38 | 1476,51 | 1484,74 | 1521,77 | 1646,71 |
| MtF14 | 924,00 | 997,96 | 1003,74 | 1112,80 | 1159,90 | 1145,57 | 1128,56 | 1091,43 |
| MtF15 | 1012,76 | 952,76 | 1123,44 | 1030,24 | 1155,35 | 1047,39 | 1167,66 | 1064,35 |
| MtF16 | 1074,73 | 1246,97 | 1263,83 | 1280,15 | 1578,81 | 1374,55 | 1474,63 | 1534,27 |
| MtF17 | 1079,72 | 960,79 | 1204,63 | 1077,10 | 1158,59 | 1096,82 | 1265,30 | 1137,75 |
| MtF18 | 1099,18 | 1109,00 | 1182,34 | 1414,99 | 1511,04 | 1322,90 | 1413,81 | 1395,86 |
| MtF19 | 1268,74 | 1005,05 | 1237,41 | 1151,53 | 1135,10 | 1156,63 | 1259,00 | 1232,07 |
| MtF20 | 814,58 | 986,89 | 870,62 | 1038,22 | 1001,67 | 1086,64 | 1098,60 | 1022,38 |
| MtF23 | 1474,28 | 1455,56 | 1895,41 | 1627,72 | 1659,34 | 1804,12 | 1958,48 | 1864,70 |
| MtF24 | 1001,84 | 1033,09 | 1070,75 | 1147,19 | 1268,19 | 1159,74 | 1464,84 | 1221,38 |
| MtF25 | 1336,46 | 1419,09 | 1495,02 | 1551,65 | 1642,04 | 1510,29 | 2313,39 | 1872,76 |
| MtF26 | 1321,48 | 1374,70 | 1548,86 | 1651,00 | 1803,86 | 1649,55 | 2004,45 | 1747,18 |
| MtF27 | 1562,54 | 1603,08 | 1469,17 | 1704,37 | 1540,18 | 1656,63 | 1770,24 | 1680,25 |
| MtF28 | 946,00 | 963,18 | 1106,98 | 1071,32 | 1173,17 | 1108,97 | 1422,22 | 1180,61 |
| MtF29 | 1209,25 | 1312,57 | 1329,88 | 1413,72 | 1540,89 | 1437,82 | 1638,09 | 1448,24 |
| MtF30 | 1204,97 | 1204,26 | 1288,67 | 1379,80 | 1482,18 | 1311,33 | 1490,67 | 1378,41 |
| MtF31 | 1072,61 | 1120,61 | 1218,04 | 1281,19 | 1364,53 | 1192,69 | 1426,44 | 1332,07 |
| MtF32 | 881,41 | 888,25 | 1042,22 | 1090,73 | 1180,04 | 1114,81 | 1489,46 | 1122,28 |
| MtF33 | 930,32 | 823,96 | 962,61 | 976,91 | 1090,55 | 1014,07 | 1257,66 | 1218,54 |
| MtF34 | 1332,62 | 1234,55 | 1423,21 | 1337,35 | 1616,61 | 1335,44 | 1604,22 | 1453,79 |

**Table S7:** Data points used to determine averages and summary statistics in Table 2 for discrimination sensitivity (d-prime) and answering bias (log ß) in response to male and female voices of the different morphing steps (0, 2, 4, 6 semitones). Participants: Cm=Control male, Cf=control female, MtF=male-to-female gender dysphoric individual.

| Participant | d-prime 0 | d-prime 2 | d-prime 4 | d-prime 6 | Log ß 0 | Log ß 2 | Log ß 4 | Log ß 6 |
| --- | --- | --- | --- | --- | --- | --- | --- | --- |
| Cm01 | 3,96 | 2,68 | 1,33 | ,14 | -,58 | -1,33 | -1,11 | -,15 |
| Cm02 | 3,63 | 2,68 | 1,90 | ,44 | 1,14 | 1,33 | ,63 | ,13 |
| Cm03 | 4,26 | 3,34 | 1,59 | ,44 | ,00 | ,56 | -,27 | -,13 |
| Cm04 | 3,96 | 3,10 | 1,50 | -,32 | -,58 | -1,80 | -1,13 | ,22 |
| Cm05 | 3,96 | 3,24 | ,76 | -,30 | -,58 | -1,65 | -,35 | ,17 |
| Cm06 | 4,26 | 3,41 | 1,59 | ,00 | ,00 | 1,44 | -,27 | ,00 |
| Cm07 | 3,96 | 3,24 | 1,75 | ,47 | -,58 | -1,65 | -1,09 | -,23 |
| Cm08 | 4,26 | 3,96 | 1,18 | -,44 | ,00 | -,58 | -,30 | ,13 |
| Cm09 | 3,34 | 2,47 | 1,15 | ,17 | -,56 | ,66 | -,06 | ,00 |
| Cm10 | 3,12 | 2,36 | 1,49 | -,17 | -,86 | 1,54 | ,33 | -,04 |
| Cm11 | 3,96 | 3,96 | 1,09 | ,18 | ,58 | -,58 | -,32 | -,08 |
| Cm12 | 4,26 | 2,76 | 1,54 | ,09 | ,00 | ,33 | -,52 | -,04 |
| Cm13 | 4,26 | 2,47 | 1,37 | -,12 | ,00 | -,66 | -,82 | ,03 |
| Cm14 | 3,41 | 2,38 | 1,37 | -,44 | 1,44 | 2,23 | ,82 | -,33 |
| Cm15 | 3,60 | 3,10 | ,84 | -1,01 | -1,14 | -1,80 | -,46 | ,59 |
| Cm17 | 3,67 | 2,97 | 1,38 | -,68 | ,00 | ,00 | -,02 | ,17 |
| Cm18 | 3,62 | 2,55 | ,84 | -1,07 | -1,11 | -1,39 | -,56 | 1,03 |
| Cm19 | 3,96 | 3,34 | 1,73 | -,59 | ,58 | ,56 | -,42 | ,03 |
| Cm20 | 3,95 | 3,07 | 1,45 | ,00 | ,61 | -1,82 | -,81 | ,00 |
| Cm21 | 3,61 | 3,34 | 1,05 | ,15 | -1,16 | ,56 | -,46 | -,06 |
| Cf01 | 3,96 | 3,41 | 1,15 | -,25 | ,58 | -1,44 | -,06 | -,01 |
| Cf02 | 3,63 | 2,36 | 1,28 | ,41 | 1,14 | 1,54 | ,82 | ,26 |
| Cf03 | 3,96 | 2,95 | 1,46 | ,00 | ,58 | 1,07 | ,00 | ,00 |
| Cf04 | 3,67 | 2,95 | 2,68 | ,36 | ,00 | 1,07 | 1,33 | ,12 |
| Cf05 | 3,96 | 2,68 | 1,92 | -,38 | ,58 | 1,33 | 1,68 | -,35 |
| Cf06 | 3,96 | 2,86 | 1,50 | -,22 | ,58 | 2,00 | 1,13 | -,16 |
| Cf07 | 3,63 | 2,39 | 1,62 | ,00 | -1,14 | ,20 | ,76 | ,00 |
| Cf08 | 3,96 | 3,41 | 2,25 | ,46 | ,58 | 1,44 | ,35 | ,18 |
| Cf09 | 3,96 | 2,95 | 1,27 | ,00 | ,58 | 1,07 | -,26 | ,00 |
| Cf10 | 3,96 | 2,80 | 1,15 | ,25 | ,58 | 1,21 | ,06 | ,01 |
| Cf11 | 2,97 | 2,21 | ,98 | -,53 | 1,91 | 2,26 | ,99 | -,66 |
| Cf13 | 3,41 | 2,56 | 1,36 | -,22 | 1,44 | 2,17 | ,58 | -,16 |
| Cf14 | 4,26 | 2,20 | 1,11 | -,09 | ,00 | ,02 | -,62 | ,03 |
| Cf15 | 2,97 | 2,47 | 1,75 | ,14 | 1,91 | 2,21 | 1,68 | ,15 |
| Cf16 | 3,95 | 2,95 | 1,54 | -,44 | ,55 | 1,93 | ,52 | -,13 |
| Cf17 | 3,96 | 3,24 | 1,98 | -,23 | ,58 | 1,65 | ,57 | -,07 |
| Cf18 | 4,26 | 3,63 | 1,54 | -,18 | ,00 | -1,14 | -,52 | ,06 |
| Cf19 | 3,96 | 2,86 | 1,54 | ,18 | ,58 | 2,00 | ,11 | ,06 |
| Cf20 | 3,10 | 2,75 | 1,07 | ,10 | 1,80 | 2,07 | ,62 | ,04 |
| MtF01 | 3,67 | 3,24 | 1,49 | -,25 | ,00 | 1,65 | ,33 | -,03 |
| MtF02 | 4,26 | 3,96 | 2,08 | ,34 | ,00 | ,58 | ,15 | ,03 |
| MtF03 | 2,95 | 2,86 | 1,37 | ,34 | 1,07 | 2,00 | ,22 | -,03 |
| MtF04 | 3,96 | 3,34 | 1,15 | -,25 | -,58 | -,56 | ,06 | ,01 |
| MtF05 | 3,63 | 2,80 | 1,14 | ,19 | 1,14 | 1,21 | ,45 | ,10 |
| MtF06 | 3,63 | 2,39 | 1,70 | -,17 | 1,14 | ,20 | -,20 | ,03 |
| MtF07 | 3,63 | 2,95 | 1,53 | ,46 | 1,14 | 1,07 | ,79 | ,18 |
| MtF08 | 3,63 | 3,63 | 1,49 | ,08 | 1,14 | 1,14 | ,33 | ,00 |
| MtF09 | 2,75 | 2,47 | 1,96 | ,38 | 2,07 | 2,21 | . | ,35 |
| MtF10 | 2,86 | 2,46 | 1,28 | ,09 | 2,00 | 1,49 | ,60 | ,04 |
| MtF11 | 3,34 | 1,95 | 1,07 | ,36 | -,56 | -,26 | -,21 | -,12 |
| MtF12 | 3,34 | 2,61 | 1,05 | ,00 | ,56 | ,51 | ,10 | ,00 |
| MtF13 | 3,34 | 2,68 | ,79 | -,46 | -,56 | -1,33 | -,18 | ,18 |
| MtF14 | 3,41 | 3,10 | 1,53 | ,18 | 1,44 | 1,80 | ,79 | ,06 |
| MtF15 | 3,96 | 2,80 | ,80 | -,77 | -,58 | -1,21 | -,45 | ,56 |
| MtF16 | 4,26 | 2,78 | 1,36 | -,09 | ,00 | -,31 | -,58 | ,04 |
| MtF17 | 3,34 | 1,35 | 1,16 | -,25 | ,56 | ,07 | -,17 | ,01 |
| MtF18 | 4,26 | 3,41 | 1,18 | ,00 | ,00 | 1,44 | -,30 | ,00 |
| MtF19 | 3,34 | 2,12 | 1,35 | -,61 | ,56 | -,47 | -,07 | ,13 |
| MtF20 | 3,24 | 2,86 | 1,45 | ,00 | 1,65 | 2,00 | ,81 | ,00 |
| MtF23 | 1,81 | 1,46 | 1,14 | -,44 | ,68 | ,16 | ,45 | -,13 |
| MtF24 | 3,96 | 2,65 | 1,25 | -,32 | ,58 | 2,13 | ,13 | -,22 |
| MtF25 | 3,24 | 3,34 | 1,49 | ,34 | 1,65 | -,56 | -,33 | -,06 |
| MtF26 | 3,63 | 3,34 | 1,68 | ,77 | 1,14 | ,56 | ,00 | -,03 |
| MtF27 | 4,26 | 3,12 | 1,14 | ,18 | ,00 | ,86 | ,45 | ,06 |
| MtF28 | 4,26 | 3,24 | 1,73 | ,51 | ,00 | 1,65 | -,42 | -,09 |
| MtF29 | 3,96 | 2,78 | 1,14 | ,09 | -,58 | -,31 | -,45 | -,03 |
| MtF30 | 3,00 | 3,34 | ,77 | -,36 | ,00 | ,56 | -,03 | ,12 |
| MtF31 | 3,63 | 3,12 | 1,31 | ,17 | 1,14 | -,86 | -,41 | -,04 |
| MtF32 | 4,26 | 3,12 | 1,90 | ,81 | ,00 | ,86 | ,63 | ,26 |
| MtF33 | 4,26 | 3,41 | 1,90 | ,39 | ,00 | 1,44 | ,63 | ,21 |
| MtF34 | 3,63 | 3,41 | 2,56 | 1,11 | -1,14 | -1,44 | -1,42 | -,81 |

**Reference:**

Junger J, Pauly K, Brohr S, Birkholz P, Neuschaefer-Rube C, et al. (2013).

Sex matters: Neural correlates of voice gender perception. Neuroimage 79: 275-287.
